# Conservation and divergence of small RNA pathways and microRNAs in land plants

**DOI:** 10.1186/s13059-017-1291-2

**Published:** 2017-08-23

**Authors:** Chenjiang You, Jie Cui, Hui Wang, Xinping Qi, Li-Yaung Kuo, Hong Ma, Lei Gao, Beixin Mo, Xuemei Chen

**Affiliations:** 1 Guangdong Provincial Key Laboratory for Plant Epigenetics, Longhua Institute of Innovative Biotechnology, College of Life Sciences and Oceanography, Shenzhen University, Shenzhen, Guangdong 518060 People’s Republic of China; 2 Key Laboratory of Optoelectronic Devices and Systems of Ministry of Education and Guangdong Province, College of Optoelectronic Engineering, Shenzhen University, Shenzhen, Guangdong 518060 People’s Republic of China; 3 Department of Botany and Plant Sciences, Institute of Integrative Genome Biology, University of California, Riverside, CA 92521 USA; 4 Shenzhen Key Laboratory of Southern Subtropical Plant Diversity, Fairylake Botanical Garden, Shenzhen & Chinese Academy of Sciences, Shenzhen, Guangdong 518004 People’s Republic of China; 5 Ministry of Education Key Laboratory of Biodiversity Sciences and Ecological Engineering and Collaborative Innovation Center for Genetics and Development, Institute of Plant Biology, School of Life Sciences, Fudan University, Shanghai, 200433 People’s Republic of China; 6 Institute of Ecology and Evolutionary Biology, National Taiwan University, Taipei, 10617 Taiwan; 7 Howard Hughes Medical Institute, University of California, Riverside, 92521 CA USA

**Keywords:** Small RNA, Lycophyte, Fern, miRNA, Evolution, Argonaute, DICER-LIKE, RdDM

## Abstract

**Background:**

As key regulators of gene expression in eukaryotes, small RNAs have been characterized in many seed plants, and pathways for their biogenesis, degradation, and action have been defined in model angiosperms. However, both small RNAs themselves and small RNA pathways are not well characterized in other land plants such as lycophytes and ferns, preventing a comprehensive evolutionary perspective on small RNAs in land plants.

**Results:**

Using 25 representatives from major lineages of lycophytes and ferns, most of which lack sequenced genomes, we characterized small RNAs and small RNA pathways in these plants. We identified homologs of DICER-LIKE (DCL), ARGONAUTE (AGO), and other genes involved in small RNA pathways, predicted over 2600 conserved microRNA (miRNA) candidates, and performed phylogenetic analyses on small RNA pathways as well as miRNAs. Pathways underlying miRNA biogenesis, degradation, and activity were established in the common ancestor of land plants, but the 24-nucleotide siRNA pathway that guides DNA methylation is incomplete in sister species of seed plants, especially lycophytes. We show that the functional diversification of key gene families such as DCL and AGO as observed in angiosperms occurred early in land plants followed by parallel expansion of the AGO family in ferns and angiosperms. We uncovered a conserved AGO subfamily absent in angiosperms.

**Conclusions:**

Our phylogenetic analyses of miRNAs in bryophytes, lycophytes, ferns, and angiosperms refine the time-of-origin for conserved miRNA families as well as small RNA machinery in land plants.

**Electronic supplementary material:**

The online version of this article (doi:10.1186/s13059-017-1291-2) contains supplementary material, which is available to authorized users.

## Background

RNAs are some of the most important components of life. They not only serve as mediators between genes and proteins as depicted in the central dogma [1], but also act as direct regulators of life processes, such as in the form of ribozymes and regulatory RNAs [2, 3]. Two major classes of small regulatory RNAs, microRNAs (miRNAs) and small interfering RNAs (siRNAs), impact a multitude of biological processes in almost all eukaryotic lineages. In plants, small RNAs repress gene expression either at the transcriptional level by DNA methylation or at the posttranscriptional level via mRNA cleavage and/or translational inhibition [4].

Plant miRNAs largely act at the posttranscriptional level—they recognize target mRNAs through sequence complementarity and lead to mRNA cleavage or translational repression [5]. The basic frameworks of miRNA biogenesis, function, and turnover have been established from studies in model angiosperms [5]. A miRNA gene is transcribed by RNA polymerase II (Pol II). The primary transcript is processed by DICER-LIKE 1 (DCL1) into a miRNA/miRNA* duplex with a length of approximately 21 nucleotides (nt) [6]. The RNA binding protein HYPONASTIC LEAVES 1 (HYL1) and the zinc-finger protein SERRATE (SE) aid DCL1 in precursor processing [7–10]. The duplex is methylated by the methyltransferase HUA ENHANCER 1 (HEN1) [11]. Afterwards, one strand of the duplex associates with ARGONAUTE1 (AGO1) to form the RNA-induced silencing complex (RISC) [5], which recognizes and represses target mRNAs [12, 13]. AGO1 is a major miRNA effector, as its endonuclease activity is responsible for target mRNA cleavage [14, 15]. HEN1 SUPPRESSOR1 (HESO1) and RNA URIDYLYLTRANSFERASE1 (URT1) are nucleotidyl transferases that act cooperatively to 3′ oligouridylate unmethylated miRNAs, thereby triggering miRNA decay [16]. SMALL RNA DEGRADING NUCLEASE (SDN) is a family of 3′-5′ exonucleases that also participate in miRNA degradation [17].

One main function of endogenous siRNAs in plants is RNA-directed DNA methylation (RdDM), which silences transposable elements and repeat sequences to maintain genome stability [4, 18]. In *Arabidopsis*, the core of this pathway is 24-nt siRNAs, usually derived from transposable elements and repeats. RNA polymerase IV (Pol IV) produces single-stranded RNAs from RdDM target loci, the RNAs are converted to double-stranded RNAs by RNA-DEPENDENT RNA POLYMERASE 2 (RDR2), and the double-stranded RNAs are processed by DCL3 to 24-nt siRNA duplexes, which undergo HEN1-mediated methylation [19, 20]. The mature siRNA is loaded into AGO4 and interacts with scaffold RNAs transcribed at the target loci by RNA polymerase V (Pol V); this interaction guides DNA methylation (mainly the CHH type where H represents any nucleotide but G) to result in the silencing of the target loci [19, 20].

Angiosperms (flowering plants), from which most of our knowledge of small RNAs and small RNA machinery has been gained, represent only one of the major land plant lineages, which include both vascular plants and non-vascular plants. There are three major types of non-vascular plants—mosses, liverworts and hornworts—which are collectively called bryophytes; they are simpler than vascular plants in morphology. Vascular plants have evolved water- and nutrient-conducting vascular tissues, which have made them much less dependent on surface water and more widely distributed to diverse terrestrial ecosystems. The dominant group of vascular plants are seed plants, including gymnosperms and angiosperms, whereas lycophytes and ferns are the closest sister groups of seed plants [21]. As sister groups of seed plants, lycophytes and ferns may help us to understand the evolutionary origins and diversification histories of the small RNA machinery and miRNAs [22]. In particular, the closest relatives to seed plants are ferns, which are also much more diverse than lycophytes; therefore, information from ferns provides an important reference for comparison with angiosperms and gymnosperms. However, only one lycophyte, *Selaginella moellendorffii*, has a sequenced genome, and none of the fern genomes has been sequenced, making it difficult to include them in comparative and evolutionary studies and limiting our understanding.

Most evolutionary studies on small RNA pathways in land plants rely heavily or exclusively on the bryophyte *Physcomitrella patens* and the lycophyte *S. moellendorffii* as representative sister groups of seed plants [23–26]. While these studies clearly reveal the existence of small RNA machinery in the common land plant ancestor, the paucity of sister groups of seed plants in these studies precluded a comprehensive picture of small RNA pathway evolution in land plants. For example, in angiosperms, *DCL* and *AGO* genes have diversified into four and three clades, respectively, representing functional diversification of small RNA pathways [25, 26]. Without information from ferns, the closest sister group of seed plants, the timing and patterns of small RNA pathway diversification were unknown or inconclusive [4, 26, 27]. Examples include the timing of the DCL2/4 divergence, AGO family expansion, and distinction between Pol IV and Pol V. Plant-specific Pol IV and Pol V are derived from Pol II with innovations in the usage of novel subunits. Some subunits that distinguish Pol IV/V from Pol II, namely the first, second, and seventh, are present in *P. patens* and *S. moellendorffii*, indicating early divergence from Pol II [24]. But the divergence between Pol IV and Pol V in land plants is more ambiguous. Based on the presence of proteins with domain structures similar to the largest subunits of Pol IV and Pol V in *P. patens*, it was concluded that Pol IV and Pol V diverged in the most recent common ancestor of land plants. But genes encoding distinct largest subunits of Pol IV and Pol V are not found in the lycophyte *S. moellendorffii* [24].

In recent years, with the development of high throughput sequencing technology, numerous miRNAs have been identified in various plant species, ranging from green algae to angiosperms. In miRBase v21 (http://www.mirbase.org), over 8000 miRNAs from 73 plant species are included. This information led to the deduction of sets of conserved miRNA families in angiosperms, seed plants, and land plants [28]. However, owing to lagging genome sequencing, the information on lycophyte and fern miRNAs is limited. The only whole-genome-sequenced lycophyte, *S. moellendorffii*, shows a miRNA profile different from that in angiosperms [29]. More recently, a number of conserved miRNAs in the fern *Pleopeltis minima* have been discovered using bioinformatic prediction methods [30]. Although comparative studies with existing datasets led to important revelations about the evolution of miRNAs and miRNA–target relationships [31–35], the limited knowledge of miRNAs from major vascular plant lineages hinders a comprehensive view of land plant miRNA evolution.

In order to better understand the molecular evolution of small RNA pathways as well as miRNAs themselves in land plants, we generated and analyzed transcriptomes and small RNAomes of lycophytes and ferns, as well as the transcriptome of the hornwort *Folioceros fuciformis* (a bryophyte). Four species representing all three orders of lycophytes and 21 species representing 11 orders of ferns were included. We identified gene families encoding major components of the small RNA machinery (biogenesis, degradation, and mode of action) from these sister species of seed plants. This allowed us to perform phylogenetic analyses on the small RNA machinery with representatives from all major land plant lineages (three green algae, three bryophytes, four lycophytes, 21 ferns, and selected seed plants). These analyses confirmed the existence of both posttranscriptional and transcriptional gene silencing pathways in ancient land plants, but more importantly, they provided unprecedented insights into the diversification of small RNA pathways in land plant evolution. The three major AGO clades found in angiosperms are present in bryophytes, lycophytes and ferns, indicating that AGO family diversification occurred early in land plant evolution. Intriguingly, we found an AGO clade present in bryophytes, lycophytes, ferns and a gymnosperm but absent in angiosperms, implicating the presence of a novel class of sRNAs in non-angiosperm species with potentially distinct functions from those in angiosperms. With regard to RdDM, our findings agree with previous reports of early divergence of Pol IV/V from Pol II [23], but support a later distinction between Pol IV and Pol V in the common ancestor of ferns and seed plants. Analyses of miRNAs and their targets (through prediction and degradome/PARE sequencing) refined the timing of origination of conserved miRNAs and revealed conserved as well as fluid miRNA–target relationships. This study provides a comparative and genome-wide view of small RNAs and small RNA machinery in sister species of seed plants and fills a major gap in the knowledge of small RNA evolution in land plants.

## Results

### Diversification of *DCL* and *AGO* genes in land plants

**Fig. 1:**
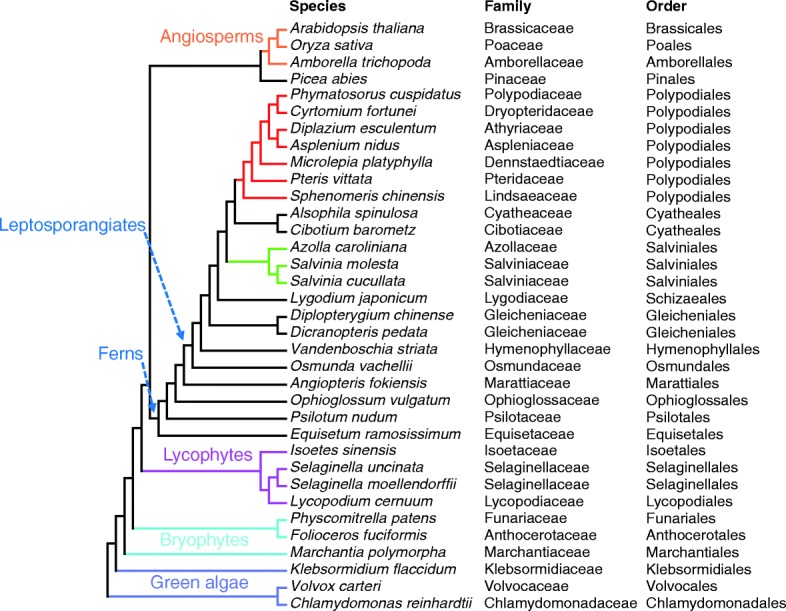
Phylogeny of green plants showing the species included in this study. Phylogeny was adopted from the new classification of extant lycophytes and ferns as described in [21]. The taxonomy (family and order) of each species is shown to the *right* of the full species names. Higher taxonomic ranks are labeled on the tree. The leptosporangiate ferns form the largest class of ferns, the Polypodiopsida. *Purple*, green algae; *light blue*, bryophytes; *magenta*, lycophytes; *red*, Polypodiales (ferns); *green*, Salviniales (ferns); *orange*, angiosperms; *black*, other ferns and gymnosperms

Previous studies showed that many genes involved in small RNA pathways are conserved in seed plants, lycophytes, and mosses [36]. Despite being the second-most species-rich group of vascular plants and the sister group of seed plants, however, ferns were not included in these studies due to a lack of available genome sequences. This limitation has hindered the understanding of the evolutionary diversification of small RNA pathways, as exemplified by the presence of distinct classes of *DCL* and *AGO* genes in angiosperms. In order to investigate components of the small RNA machinery in sister species of seed plants, we sequenced the transcriptomes of the hornwort *F. fuciformis* (a bryophyte), four species from all three orders of lycophytes, and 21 species covering all 11 orders of ferns (Fig. 1; Additional file 1: Table S1). From *de novo* assembled transcripts plus reported genes in *S. moellendorffii*, we identified, from these species, homologs of major components of the small RNA machinery in angiosperms (Additional file 1: Table S2). We first performed phylogenetic analyses with *DCL* and *AGO* genes identified from these species. We also included in the analyses *DCL* and *AGO* genes from several species that occupy key positions in land plant evolution and have sequenced genomes or transcriptomes, including two bryophytes, *P. patens* [37] (a moss) and *Marchantia polymorpha* [38] (a liverwort), the gymnosperm *Picea abies* [39], the sister species of extant angiosperms, *Amborella trichopoda* [40], and the model angiosperms *Oryza sativa* and *Arabidopsis thaliana*.

**Fig. 2:**
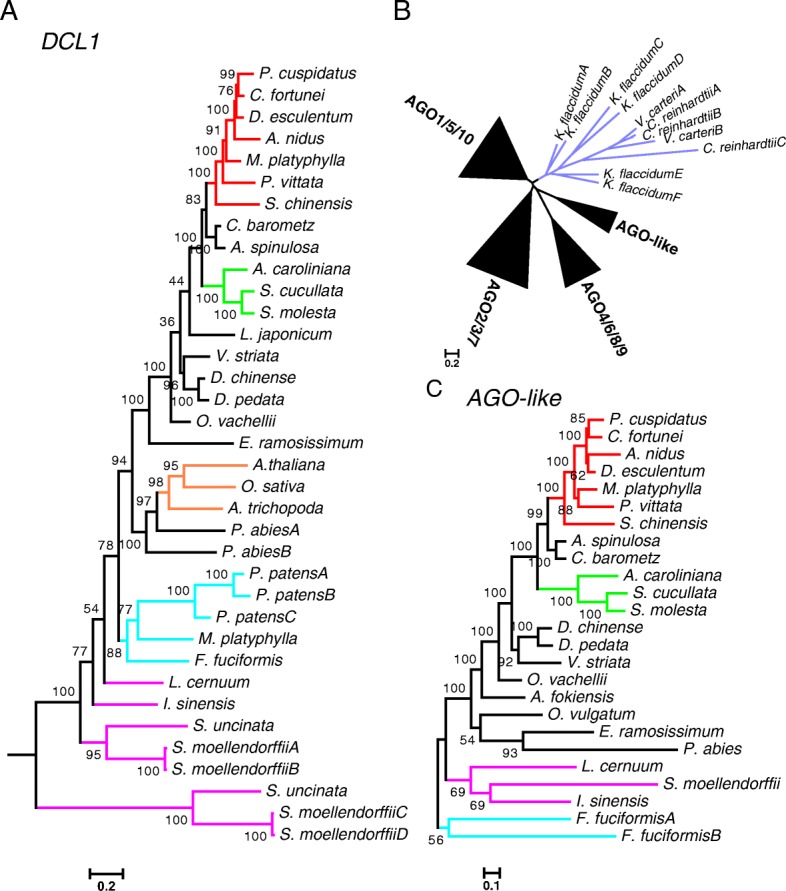
Phylogenetic trees of vital genes involved in small RNA pathways in land plants. **a** A maximun likelihood (ML) tree for *DCL1* genes in representative land plants. The other subclades of *DCL* genes (such as *DCL3* and *DCL4*) are shown in Additional file 2: Figure S1. Alignment length, 22,788 nt. **b** A schematic tree of *AGO* genes. The sequences from green algae (*Chlamydomonas reinhardtii*, *Volvox carteri*, and *Klebsormidium flaccidum*) are clustered. The *AGO* genes from land plants form four clades, three of which are named after the *AGO* genes from *A. thaliana*. **c** A ML tree for the *AGO*-like clade in **b**. Other *AGO* clades in land plants (such as *AGO1*/*5*/*10*) are shown in Additional file 2: Figure S2. *Purple*, green algae; *light blue*, bryophytes; *magenta*, lycophytes; *red*, Polypodiales (ferns); *green*, Salviniales (ferns); *orange*, angiosperms; *black*, other ferns and gymnosperms. The *scale bar* represents nucleotide substitution rates. *Numbers* beside *nodes* are bootstrap support values showing confidence (from 0 to 100). Alignment length, 3075 nt

Previous phylogenetic analyses of angiosperm *DCL* genes showed the presence of four clades, represented by *Arabidopsis DCL1–4* [26]. Our phylogenetic analyses of *DCL* genes from three bryophytes, four lycophytes, 21 ferns, and four seed plants also found four clades, but revealed differences between *DCL2* and other *DCL* genes. Orthologs of *DCL1*, *3*, and *4* were identified in each of the land plants and as a single copy in most species (Fig. 2a; Additional file 2: Figure S1; Additional file 1: Table S2), indicating early divergence of these *DCLs* and implying the functional conservation of *DCL1*, *3*, and *4* in land plants. However, homologs of *DCL2*, which generates 22-nt siRNAs in *Arabidopsis* and has been identified in many angiosperms [26], were only found in *Psilotum nudum*, *Osmunda vachellii*, and *Equisetum ramosissimum*, which are sister species of leptosporangiates, but not in any other species in this study (Additional file 1: Table S2). The lack of detection in the three bryophytes, the four lycophytes, and the other ferns could be due to their absence in these organisms. Alternatively, *DCL2* may be spatiotemporally regulated in its expression and eluded detection by RNA-seq in the tissues examined; in fact, *DCL2* is strongly induced by viral infection in angiosperms [41, 42]. Intriguingly, orthologs of *DCL2* were not found in the annotated genomes of the lycophyte *S. moellendorffii* and the moss *P. patens* (Additional file 1: Table S2), nor in the genome of the bog moss *Sphagnum fallax* [43]. Thus, *DCL2* may be absent in these sister species of euphyllophytes, raising the possibility that *DCL2* originated in the latest common ancestor of ferns and seed plants after its divergence from lycophytes.

*AGO* genes encode proteins that serve as effectors of various small RNAs and, in angiosperms, form three major phylogenetic clades that can be dated back to the most recent common ancestors of land plants, each with distinct functions through binding to different groups of small RNAs [25, 27, 44, 45]. In bryophytes, lycophytes, ferns, and one gymnosperm, the *AGO* genes formed four clades (Fig. 2b), three of which corresponded to the three clades in angiosperms (Additional file 2: Figure S2). In each of the three clades, the *AGO* genes evolved independently in each land plant lineage, such that the gene tree mimicked that of the species tree (Additional file 2: Figure S2). Thus, the AGO diversity in each of the three clades in seed plants occurred after the fern/seed plant divergence. In the AGO1/5/10 and AGO2/3/7 clades, expansion occurred in leptosporangiate ferns, the largest lineage including Polypodiales and Salviniales species [21] (Additional file 2: Figure S2a, b). Thus, one or more ancient duplications likely occurred in this lineage after splitting from Osmundales (arrowheads in Additional file 2: Figure S2). The additional clade of *AGO* genes (Fig. 2c) in the hornwort *F. fuciformis*, lycophytes, ferns, and the gymnosperm *P. abies* encoded AGO proteins containing the functional domains as well as the catalytic residues as in angiosperm AGO proteins (Additional file 2: Figure S3). The presence of AGO members of this clade in the hornwort, lycophytes, ferns, and the gymnosperm suggests an early origin of this clade but a subsequent loss in angiosperms.

### Conservation of the miRNA machinery in land plants

We identified homologs of angiosperm genes involved in miRNA biogenesis, such as *DCL1*, *SE*, and *HEN1*, in miRNA degradation, such as *HESO1*, *URT1*, and *SDN*, and in miRNA activity, such as *AGO1*, from bryophytes, lycophytes, and ferns (Additional file 1: Table S2). *DCL1* encoding the major enzyme in miRNA biogenesis was conserved in all land plant lineages analyzed (Fig. 2a). The *DCL1* orthologs in bryophytes, lycophytes, and ferns formed a monophyletic group with the *DCL1* genes in seed plants (Additional file 2: Figure S1), separate from other *DCL* clades, indicating early divergence of the miRNA pathway from siRNA pathways in land plants. The *AGO1* gene belongs to the *AGO1*/*5*/*10* clade in angiosperms [14, 27]. The aforementioned phylogenetic analyses of *AGO* genes in bryophytes, lycophyes, ferns, and seed plants indicated that the *AGO1*/*5*/*10* clade originated in the most recent common ancestor of land plants (Additional file 2: Figure S2a). Phylogenetic analyses with homologs of *SE*, *HEN1*, *HESO1*, *URT1*, and *SDN* showed that homologs from all land plants formed a monophyletic group for each of the genes (Additional file 2: Figure S4), suggesting that each gene existed in the most recent common ancestor of land plants. Homologs of *HEN1*, *HESO1*, and *URT1* were found as single copy in each bryophyte, lycophyte, and fern species, raising the possibility that they are orthologs (Additional file 2: Figure S4a, d), whereas those of *SE* had multiple copies in some of the species (Additional file 2: Figure S4c). Together, these findings suggest that the mechanisms of miRNA biogenesis and degradation were established in the most recent common ancestor of land plants.

The unicellular green alga *Chlamydomonas reinhartii* has miRNAs [46, 47]. This raises the question of whether land plant miRNA pathways already existed in the common ancestor of green plants. We searched for homologs of *DCL1*, *SE*, *HEN1*, *AGO1*, *SDN*, *HESO1*, and *URT1* in *C. reinhardtii* [48] and two multicellular green algae, *Volvox carteri* [49] and *Klebsormidium flaccidum* [50]. Algal homologs for all genes except for *SE* were found, and phylogenetic analyses showed that algal *HEN1*, *SDN*, *HESO1*, and *URT1* genes each formed a monophyletic group with their land plant homologs, implying that these genes (all encoding enzymes) may have similar molecular functions to their land plant counterparts. However, the functions of these genes in *Arabidopsis* are not restricted to miRNAs, e.g., *HEN1* also acts on siRNAs [51] and HESO1 and URT1 also act on long RNAs [52, 53]; thus, the phylogenetic conservation in these genes could not be interpreted to support the presence of a common miRNA pathway in algae and land plants.

As the diversification of *DCL* and *AGO* genes in land plants reflects the functional diversification of small RNA pathways (such as miRNA vs. siRNA), we turned to examine whether *DCL* and *AGO* diversification already occurred in the common ancestor of green plants. *AGO* genes in green algae clustered separately from those in any other plant species (Fig. 2b), suggesting that the four land plant *AGO* clades were established after the algae/land plant split. Similarly, all algal *DCL* genes were outgroups to all land plant *DCL* clades (Additional file 2: Figure S1), suggesting that *DCL* diversification occurred in the common ancestor of land plants. Thus, an ancient small RNA-based silencing pathway existed in the common ancestor of green plants (as shown by the presence of *DCL*, *AGO*, and *HEN1* genes in algae and land plants), but the land plant miRNA machinery probably became specialized after the algae/land plant split.

### The RdDM machinery was not fully established in ancient land plants

RdDM is a transcriptional gene silencing process that involves Pol IV and Pol V in angiosperms [19]. *RDR2* also acts in RdDM while its paralog in *Arabidopsis*, *RDR6*, acts in posttranscriptional gene silencing. In order to evaluate the evolution of RdDM in land plants, we identified genes encoding key subunits of each of the five RNA polymerases (Pol I, II, III, IV, and V), as well as *RDR* genes in green algae, bryophytes, lycophytes, ferns, and seed plants, and performed phylogenetic analyses (Additional file 2: Figure S5). In land plants, two separate monophyletic groups, *RDR1*/*2* and *RDR6*, were found, indicating that *RDR1*/*2* and *RDR6* diverged from each other early in land plants (Additional file 2: Figure S5d) and suggesting the existence of both transcriptional and posttranscriptional gene silencing in ancient land plants. Homologs of *RDR* genes in green algae clustered exclusively from those in all examined land plants, suggesting that the *RDR* diversification into the posttranscriptional and transcriptional gene silencing roles occurred after the algae/land plant split.

To evaluate the divergence of Pol IV/Pol V from Pol II, we examined the phylogeny of the second, fourth, and seventh subunits that are specific to, and shared by, Pol IV and Pol V in angiosperms [23]. *NRPA2*/*B2*/*C2*, encoding the second largest subunit of Pol I, II, and III, respectively, formed three monophyletic groups, respectively, showing that they are distinct from one another and extremely conserved across green plant lineages. Homologs of *NRPD2* were found in almost all bryophytes, lycophytes, and ferns examined (Additional file 1: Table S2), and the phylogeny of the genes showed early divergence from *NRPB2* in land plant evolution (Additional file 2: Figure S5b). Similarly, *NRPD7* from all land plants examined formed a monophyetic group separate from that of *NRPB7* (Additional file 2: Figure S5c). Together, the *NRPD2* and *NRPD7* phylogenies indicate that a Pol IV- or Pol V-like polymerase diverged from Pol II in the most recent common ancestor of land plants. However, we did not detect any *NRPD4* genes in any of the bryophyte, lycophyte, fern or gymnosperm species, suggesting that this subunit of Pol IV/V evolved specifically in angiosperms.

**Fig. 3:**
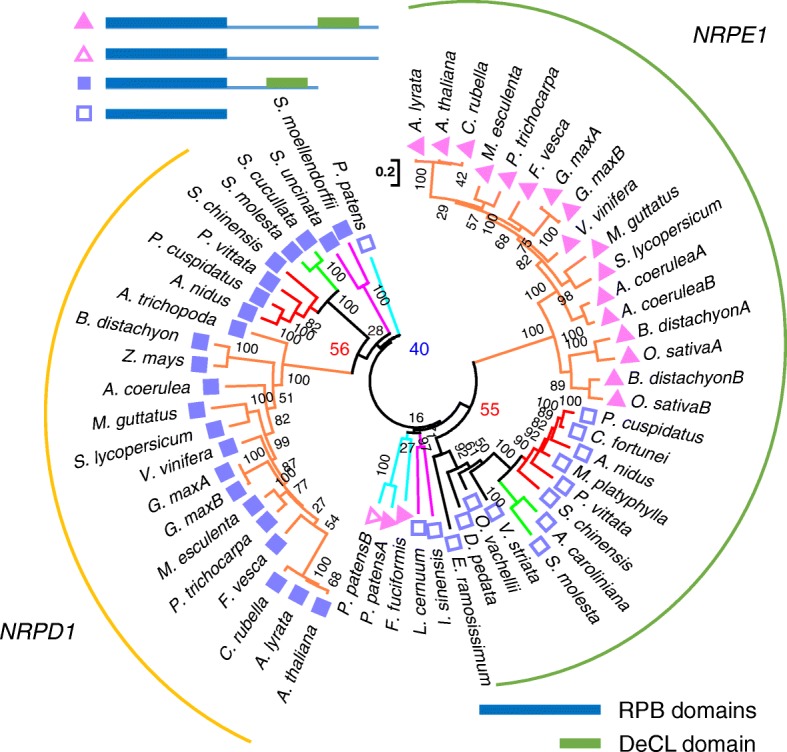
A phylogenetic tree of *NRPD1*/*E1* in land plants. A maximun likelihood (ML) tree for *NRPD1*/*E1* is shown in a radial format. Sequences in bryophytes, lycophytes, and ferns were obtained from assembled transcripts in this study, and sequences in angiosperms were from Phytozome. Simplified protein structures are shown beside the sequence accessions and in the *upper left corner*. RPB domains are represented by the *blue rectangles* and the DeCL domain is represented by the *green rectangles. Light blue*, bryophytes; *magenta*, lycophytes; *red*, Polypodiales (ferns); *green*, Salviniales (ferns); *orange*, angiosperms; *black*, other ferns. Numbers beside nodes are bootstrap support values showing confidence (from 0 to 100). Key values are enlarged and labeled with different colors: *red* (over 50) for the clustering of genes from ferns and angiosperms and *blue* (less than 50) for the clustering of earlier-divergent species and ferns/angiosperms. Alignment length, 1830 nt

We were particularly interested in examining the phylogeny of *NRPD1* and *NRPE1*, which encode the largest subunit of Pol IV and Pol V, respectively, as they represent the divergence of Pol IV and Pol V. Two previous studies relying largely or solely on the bryophyte *P. patens* and the lycophyte *S. moellendorffii* as the sister groups of angiosperms reached different conclusions. One study suggested separation of *NRPD1* and *NRPE1* in the most recent common ancestor of land plants [23], while the other suggested the presence of *NRPE1* in all land plants with *NRPD1* having evolved in later lineages [24]. Interestingly, unlike *NRPA1*/*B1*/*C1* (encoding the largest subunit of Pol I, II, and III, respectively), which were identified in all the bryophyte, lycophyte, and fern species examined, *NRPD1*/*E1*-like genes were only found in some of the fern species (Additional file 1: Table S2), which may be attributable to low levels of, or spatiotemporally restricted, expression of *NRPD1*/*E1* or even their absence in these species. Nevertheless, phylogenetic analyses were performed with *NRPD1*/*E1* genes from two bryophytes, four lycophytes, 13 ferns, and 14 angiosperms including *Amborella*. There was moderate support for the grouping of *NRPD1* from two orders of ferns and angiosperms, as well as for the grouping of *NRPE1* from the same ferns and angiosperms (Fig. 3). In bryophytes and lycophytes, however, the *NRPD1*/*E1* genes could not be confidently assigned to either the *NRPD1* or *NRPE1* clade (Fig. 3). Note that a single *NRPD1*/*E1*-like gene was detected in each of the four lycophytes (Fig. 3; Additional file 1: Table S2). The genes from two lycophytes (*S. moellendorffii* and *S. uncinata*) grouped with *NRPD1* from ferns/angiosperms with a bootstrap value of 28 (Fig. 3), while the genes from the other two lycophytes (*Isoetes sinensis* and *Lycopodium cernuum*) grouped with *NRPE1* from ferns/angiosperms with a bootstrap value of 16 (Fig. 3).

In addition to sequence similarity, previous studies also considered the domain structures of NRPD1 and NRPE1 to distinguish the two [24]. NRPD1 and NRPE1 in angiosperms contain an N-terminal domain similar to that in NRPB1 (RPB domain) and a C-terminal DeCL domain. The region between the two domains is long in NRPE1 and short in NRPD1 in angiosperms (Fig. 3). We found that this was not a reliable feature to distinguish the two proteins. The fern proteins that grouped with angiosperm NRPE1 with good bootstrap support did not have the DeCL domain (Fig. 3). Given the poor support for the clustering of *NRPD1*/*E1*-like genes from bryophytes and lycophytes with either *NRPD1* or *NRPE1*, we conclude that the divergence of Pol IV and Pol V probably occurred in the most recent common ancestor of ferns and seed plants. The sister group of current ferns and seed plants probably had a single Pol II derivative.

### The 24-nt small RNA class is missing in many samples

**Fig. 4:**
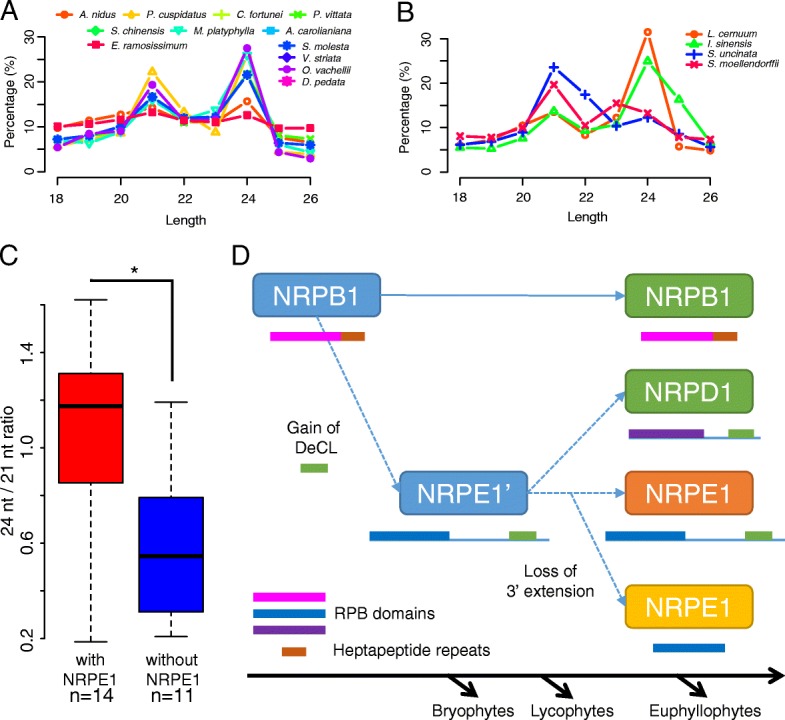
Size distribution of small RNA reads. **a** Relative proportions of small RNA reads as percentages for each size category in total reads in species with *NRPE1* as shown in Fig. 3. **b** Relative proportions of small RNA reads as percentages for each size category in total reads in four lycophytes. There are no obvious 24-nt peaks in *S. moellendorffii* (*red*) and *Selaginella uncinata* (*blue*). For **a** and **b**, the total numbers of small RNA reads for each sample are included in Additional file 1: Table S1. **c** Ratio of counts of 24- and 21-nt small RNAs. Ratios in species with *NRPE1* and without *NRPE1* are grouped and plotted, and the difference between these two groups is significant (as shown with *asterisk*). The *P* value (0.018) was determined with a Wilcoxon test. **d** Model for NRPD1/E1 origination. In the most recent common ancestor of land plants, an extra NRPB1 copy from a duplication event fused with a DeCL domain at the C-terminus and formed the ancestral NRPE1. In the most recent common ancestor of euphyllophytes, this copy duplicated and one copy lost some C-terminal sequences and formed NRPD1. The other copy retained the whole protein structure as NRPE1 in ancient seed plants, but lost the long C-terminal extension in the most recent common ancestors of ferns. RPB domains are represented by the *magenta* (NRPB1), *blue* (NRPE1), or *purple* (NRPD1) *rectangles* and the DeCL domain is represented by the *green rectangles*. Heptapeptide repeats of NRPB1 are shown as *brown rectangles*. NRPE1 is shown in *orange* for bryophytes and angiosperms and in *yellow* for lycophytes and ferns

The existence of genes encoding the small RNA machinery in lycophytes and ferns prompted us to ask whether the small RNA landscapes are similar in lycophytes, ferns, and angiosperms. We sequenced 18–26-nt small RNAs from the same species (four lycophytes and 21 ferns, but not for the hornwort *F. fuciformis* (Additional file 1: Table S1)). To enrich for miRNAs and siRNAs, reads were filtered against *S. moellendorffii* rRNA, tRNA, snRNA, and snoRNA sequences. Over 153 million reads in total were obtained for the 25 samples, one from each species (see details in Additional file 1: Table S1). We determined the size distribution of small RNAs in lycophytes and ferns. Although most lycophytes and ferns had two peaks, 21 nt and 24 nt, the relative sizes of the peaks varied (Fig. 4a; Additional file 2: Figure S6). For the ferns in Polypodiales, from which an *NRPE1* homolog was detected (Fig. 3), the 24-nt peak was equal to or larger than the 21-nt peak, as in angiosperms (Fig. 4a). For most of the other species, from which no *NRPE1* homolog was detected (Fig. 3), the 24-nt peak was less prominent (Additional file 2: Figure S6). A significant difference was found for the ratio of the 24- and 21-nt peaks between the species with detected *NRPE1* and those without detected *NRPE1* (Fig. 4c). Among the four lycophytes we sequenced, the 24-nt peak was obvious in *I. sinensis* and *L. cernuum* but not in *S. moellendorffii* and *S. uncinata* (Fig. 4b). It mirrors the previous finding in *S. moellendorffii* adult aerial tissues that CHH methylation, which is maintained by RdDM under the guidance of 24-nt siRNAs, was missing [54]. Interestingly, the two lycophyte species without a prominent 24-nt peak had an NRPD1/E1-like gene with a domain structure more like NRPD1 (Fig. 3), while the two lycophytes with a prominent 24-nt peak had an NRPD1/E1-like gene with similar domain structure as fern NRPE1 (Fig. 3).

### Identification of miRNAs that are conserved among vascular plants

Although the 24-nt peak was variable in abundance and possibly existence in lycophytes and ferns, the 21-nt peak, largely composed of miRNAs in angiosperms [31], was clearly discernable in nearly all lycophytes and ferns (Fig. 4a, b; Additional file 2: Figure S6). A gold standard to validate miRNAs in plants [55] is to interrogate the secondary structures of putative precursors. However, the lycophytes and ferns in this study have not been genome-sequenced except for *S. moellendorffii*, and the precursors of miRNAs are difficult to detect by RNA-seq because of their low abundance. Therefore, we focused on conserved miRNAs (miRNAs with sequence similarity to annotated miRNAs) as well as validated miRNAs using the genomic sequences of *S. moellendorffii*.

Previous findings in *P. patens* and *S. moellendorffii* revealed that some miRNAs were conserved with those in angiosperms [28]. To find conserved miRNAs in the lycophytes and ferns, we adopted and modified the method described in Chávez Montes et al. [28] (see “Methods” for details). Because of the existence of homologs of miRNA modification enzymes like HESO1 and URT1 in these species (Additional file 2: Figure S4d), we took 3′ truncation and tailing of miRNAs into account and combined predicted miRNA candidates with identical 1–16 nucleotides from their 5′ ends into one cluster, from which the most abundant one was used as the representative (Additional file 2: Figure S7). As a result, 2675 miRNA clusters from 258 miRNA families matching reported plant miRNAs were identified (Additional file 3; Additional file 1: Table S3).

To confirm the reliability of prediction of conserved miRNAs, we sought to validate the predicted miRNAs in genome-sequenced *S. moellendorffii*. We first mapped the miRNA candidates to the assembled transcripts from our RNA-seq datasets, and discovered nine transcripts containing hairpin structures accommodating mature miRNAs, indicating that these transcripts may be the precursors to these miRNAs (Additional file 1: Table S4). Of these nine putative precursors, seven contained annotated miRNAs, including miR1081, miR1086, miR1098, miR1105, miR1107, miR1113, and miR171c. The same transcript containing miR171c also contained miR171c*, which was detected in small RNA-seq. The remaining two transcripts supported the prediction of two new miRNAs, miR5054.2429 and miR536.2447. About 49% of the small RNAs from our small RNA-seq from *S. moellendorffii* were mappable to the *S. moellendorffii* genome. This low mapping rate might result from sequencing error or incompleteness of the genome. Among the 209 miRNA candidates identified in our study, 69 could be mapped to the genome; in addition, 41 out of the 69 mapped candidates were accommodated in hairpin structures in the genome (Additional file 1: Table S4), indicating high confidence in miRNA prediction. Among the 69 candidates, 25 were among the 64 annotated *S. moellendorffii* miRNAs in miRBase v21 (Additional file 1: Table S4). Two predicted Smo-miR164 isoforms (not among the annotated miRNAs) mapped to an unusually high number of loci in the genome (Additional file 1: Table S4) compared with other miRNAs, suggesting that these two small RNAs were likely generated from repetitive sequences and were probably siRNAs. Other predicted but not previously annotated miRNA candidates may be true miRNAs. For example, the miR6300 family mapped to genomic regions containing hairpin structures, and this miRNA was found in all four lycophytes and 21 ferns (see below).

### Features of lycophyte and fern miRNAs

#### Length

**Fig. 5:**
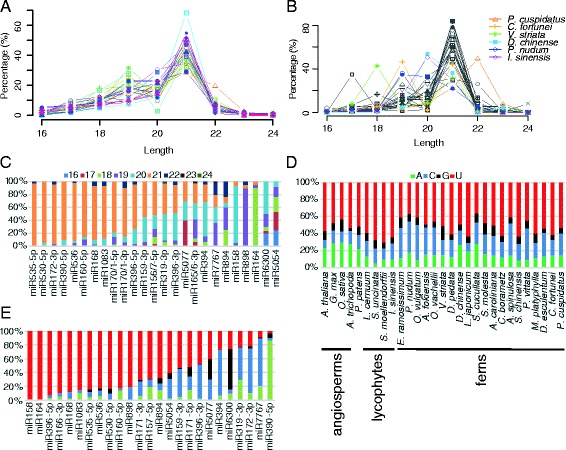
Sequence features of conserved miRNA candidates. **a** Relative proportions of miRNA candidates for each size category in all 25 species. The miRNA population sizes for each sample are shown in Table 1. **b** Relative abundance of miRNA candidates for each size category in the species in this study. Only species in which 21-nt miRNA candidates are not the most abundant are labeled. The population sizes for each sample are included in Additional file 1: Table S1. **c** Length distributions of miRNA candidates shown as percentages in each conserved miRNA family. The miRNAs of angiosperms and *P. patens* are from miRBase v21 (http://www.mirbase.org/). **d**, **e** The 5′ nucleotide composition of miRNA candidates shown as percentages of each of the four nucleotides. **d** The 5′ nucleotide composition of miRNA candidates in 25 lycophytes and ferns in this study and annotated miRNAs from five other species. All miRNA candidates or annotated miRNAs from each species were included in the analysis. **e** The 5′ nucleotide composition of conserved miRNA families from lycophytes and ferns in this study. miRNA candidates from all species from which the miRNA candidates were detected are included. Sequences of all predicted miRNA candidates in lycophytes and ferns are included in Additional file 4

Similar to angiosperm miRNAs, the majority of miRNA candidates from the studied species were 21 nt long (Fig. 5a). However, in several species, including *Phymatosorus cuspidatus*, *Cyrtomium fortunei*, *Vandenboschia striata*, *Diplopterygium chinense*, *P. nudum*, and *I. sinensis*, the most abundantly expressed ones were not 21 nt, but 18 to 20 nt or 22 nt long (Fig. 5b). The most abundant miRNA in *P. cuspidatus* was the 22-nt miR1511.1748 (one isoform of miR1511), which was similar to miR1511 in *Malus X domestica* [56]. However, this small RNA was identical to nucleotides 1–22 from a 24-nt small RNA that matches a reported copia-like LTR-retrotransposon in monocots [57], indicating that this miRNA candidate may be an siRNA generated from repetitive sequences. According to the spontaneous model of the origin of miRNAs, a proto-*MIR* gene is first processed by DCLs to generate siRNAs; then further evolution by point mutations and gain of function result in a true miRNA precursor suitable for DCL1 processing [58]. Perhaps this miRNA candidate is still evolving to be a genuine miRNA in *P. cuspidatus*. To determine the length of miRNAs in individual miRNA families, we focused on the top 25 most conserved miRNA families, defined as being detected in more than 15 of the lycophytes and ferns examined. Consistent with the size distribution of the total miRNA population, the predominant length of these conserved miRNA candidates was also 21 nt (Fig. 5c), which is also the predominant length of miRNAs in angiosperms [59]. However, members of some miRNA families differed from this length. For instance, most of the miR898 and miR6300 members were 19- or 20-nt long, and most miR164s were only 18-nt long (Fig. 5c). Although we found some 23- or 24-nt long miRNA candidates, almost all members of each conserved family were shorter than 22 nt. The similar length of conserved miRNAs in land plants probably reflects similar mechanisms of precursor recognition/processing by DCL1 in land plants, whereas some miRNAs with shorter lengths suggest possible differences in the miRNA mechinary in ferns, an idea also supported by the additional clade of *AGO* genes in ferns and non-angiosperm plants.

#### The 5′ nucleotide

Because the nature of the 5′ nucleotide of small RNAs is a critical feature relevant to their sorting into different AGOs [60], we examined the 5′ nucleotide identity of these miRNA candidates. In all lycophyte and fern species, the most prevalent 5′ nucleotide of miRNA candidates was U (Fig. 5d), which is also the most prevalent 5′ nucleotide in angiosperm miRNAs [61], a feature of miRNAs that is probably related to AGO1’s preference for 5′ U [14]. Interestingly, the second most prevalent 5′ nucleotide in lycophyte and fern miRNA candidates was C, as opposed to A in angiosperms (Fig. 5d). In addition, by comparing the 5′ U prevalence between ferns and lycophytes and between lycophytes and other land plants (*P. patens*, *A. trichopoda*, *O. sativa*, *Glycine max*, and *A. thaliana*), we found that 5′ U was significantly more prevalent in lycophytes than in ferns (*P* value < 3.87e-11, χ^2^ test) or in other plants (*P* value < 6.17e-12, χ^2^ test). This implicates different AGO sorting patterns in lycophytes. To determine the 5′ nucleotide identity in individual miRNA families, we also focused on the 25 most conserved miRNA families mentioned above. The identities of the 5′ nucleotides were different among these miRNA families (Fig. 5e). For example, over half of miR6300-3p family members had a 5′ G, and the majority of miR7767 had a 5′ C, which were consistent with the situations in soybean and *Brachypodium distachyum*, respectively [62, 63]. However, the 5′ C prevalence in miR394s in lycophytes and ferns is not found in the currently annotated miR394s in angiosperms (miRBase v21), suggesting an early divergence of miR394 in land plants.

#### *Isoforms and*/*or sequence variations*

**Fig. 6:**
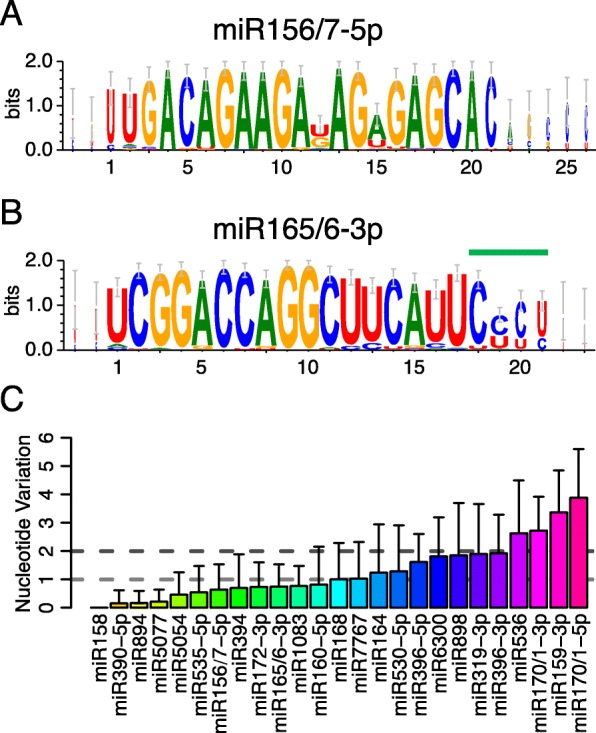
Conservation of miRNA candidates in conserved miRNA families. **a**, **b** Sequence logos showing the consensus sequences of miR156/157-3p and miR165/166-3p families from lycophytes and ferns. The overall height of each position indicates the conservation at this position (in bits), and the height of each nucleotide shows the relative frequency of this nucleotide at this position. In the miR165/166-3p family, nucleotides 19–21 (marked by the *green line*) are not as conserved as the 5′ end; this is probably because of 3′ truncation and U tailing. **c** Barplot for nucleotide variations in conserved miRNA families. Only the top one or two most abundant miRNAs in each miRNA family from each species were included in the analyses. The *horizontal dotted lines* indicate the average nucleotide variations of 1 and 2, respectively, and divide these miRNA families into three groups, families with average nucleotide variations less than 1, between 1 and 2, and more than 2. Different colors in the columns indicate various miRNA families and error bars represent standard deviations of nucleotide variations in each miRNA family

Because our miRNA identification was based on sequence similarity, some miRNA families reported in angiosperms were detected in lycophytes and ferns. For example, the consensus miRNA sequences of two families in lycophytes and ferns showed few differences from those in angiosperms (Fig. 6a, b). In addition to reads that were identical to annotated miRNAs, we identified many isoforms (nucleotide variations, 3′ truncated and/or tailed species) within each species. Nucleotide variations may be attributed to sequencing error, or to true differences among alleles or paralogs in one species. In *S. moellendorffii*, we detected over a dozen miR165/6 isoforms, most of which showed one nucleotide variation and were at low abundance. Since only the form identical to the annotated miRNA matched the genome, the minor isoforms were likely attributable to sequencing error. An example of potentially true miRNA derivatives was the miR165/6-3p family, whose members identical to the annotated smo-miR166a/b/c were frequently 3′ end truncated and U-tailed (Fig. 6b; Additional file 1: Table S4), suggesting that this family was highly susceptible to degradation.

To understand further the conservation/divergence of miRNAs, we examined sequence similarities of conserved miRNA candidates in lycophytes and ferns. To minimize the effects of sequencing error, we focused only on the top one or two most abundant isoforms in each species, and calculated nucleotide variation across the lycophytes and fern species for each conserved family (defined as being detectable in over 15 species of lycophytes and ferns) by comparing to the consensus sequence (Fig. 6c). Many of these miRNA families, including miR158, miR156/7-5p, miR165/6-3p, and miR172-3p, were quite conserved, and had less than one nucleotide variation on average compared to the consensus sequence. Most of the remaining miRNA families varied by less than two nucleotides. However, four families were relatively less conserved; these were miR536, miR159-3p, miR170/1-5p, and miR170/1-3p (Fig. 6c).

### Conservation

**Fig. 7:**
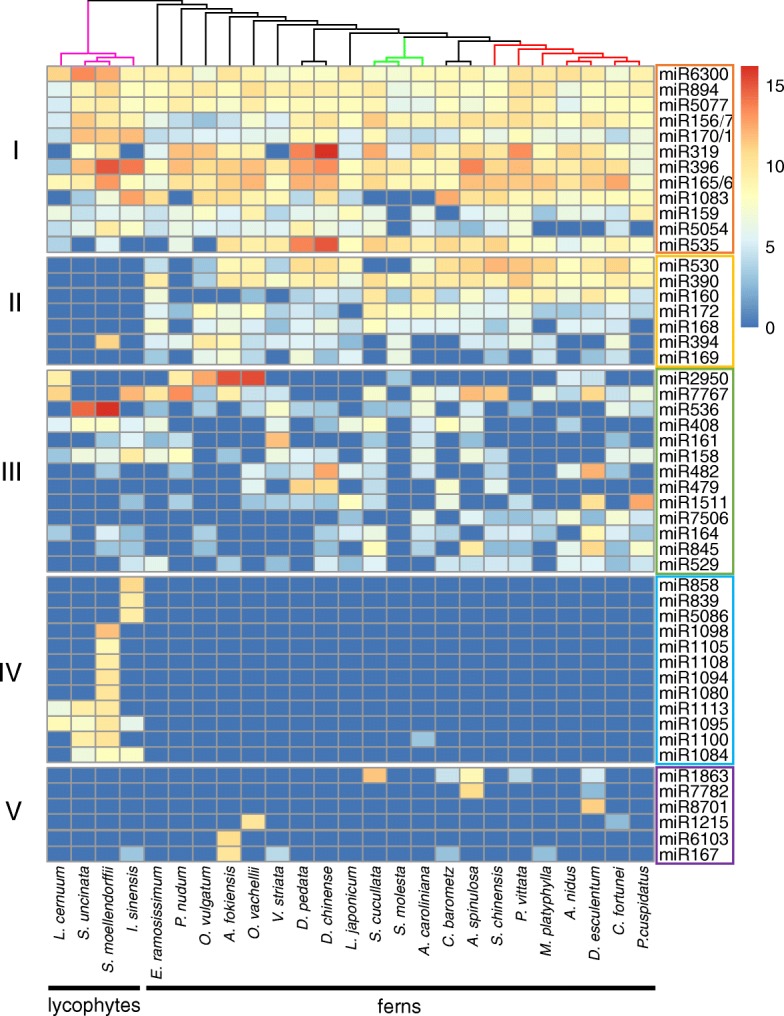
Heatmap for conserved miRNA candidates. Each row represents a miRNA family. Colors indicate the scaled expression levels of these miRNA families (log2(RPM)). Rows are divided into five groups: class I miRNA candidates were found at high levels in all species; class II miRNA candidates were found in most ferns but few or no lycophytes; class III miRNA candidates were detected at high levels in multiple species in both lycophytes and ferns; class IV miRNA candidates were detected mainly in lycophytes; and class V miRNA candidates were at high levels in specific species

A previous study reported many conserved miRNA families in land plants [28]. Families like miR156/7, miR159, miR165/6, miR172, miR390, etc. were present in all vascular plants studied, including one fern, *Marsilea quadrifolia*. The extensive miRNA data from lycophytes and ferns in this study enabled better evaluation of conserved miRNA families in various lineages. As previous studies show that conserved miRNAs tend to maintain a high level of expression [64], we integrated miRNA abundance in our analysis of miRNA conservation. We clustered the highly expressed miRNA families (top 50 in mean RPM in all species) into five groups according to their expression patterns. We found that 12 miRNA families were present in almost all examined lycophytes and ferns and had the highest abundance, forming the class I miRNA candidates (Fig. 7). The reported miR156/7, miR170/1, miR319, miR396, miR165/6, and miR159 [28] were among them. Other families such as miR6300 and miR5077 may be conserved only in lycophytes and ferns but not in other vascular plants. We also identified miRNA families which were detectable in nearly all ferns, forming class II. It is intriguing that in lycophytes, several miRNAs that are conserved and important in vascular plants (class II), including miR168 (targeting *AGO1*) [65] and miR172 (targeting *AP2*) [66], were not found (Fig. 7), suggesting either restricted expression precluding their detection or the absence of these miRNAs in lycophytes. We found some miRNAs (class IV) to be lycophyte-specific.

#### phasiRNA triggers

Phased siRNAs (phasiRNAs), including *trans*-acting siRNAs (ta-siRNAs), are a class of secondary siRNAs, the biogenesis of which is triggered by the cleavage of target transcripts (from *PHAS* and *TAS* genes) by a few specific miRNAs [67, 68]. Known phasiRNA-triggering miRNAs in angiosperms include miR161 targeting *PPR* genes, miR168 targeting *AGO1*, miR173 targeting *TAS1* and *TAS2*, miR390 targeting *TAS3*, miR393 targeting F-box genes, and miR828 targeting *TAS4* in *Arabidopsis* [67], and others such as miR4392 in soybean [69], and miR2118 and miR2275 in monocots [70]. We searched for homologs of the angiosperm phasiRNA-triggering miRNAs in lycophytes and ferns. miR161 was detected in nine species of lycophytes and ferns (Fig. 7; Additional file 1: Table S3). Interestingly, some phasiRNA-triggering miRNAs were detected in ferns but not in lycophytes, including miR168 and miR390 (Fig. 7; Additional file 1: Table S3). It is thought that miR390 is conserved in land plants, as in the moss *P. patens*, which is the sister group of vascular plants, miR390 targets *TAS* to generate secondary siRNAs [68, 71]. To further shed light on phasiRNA evolution, we tried to identify phased siRNA clusters in the lycophyte *S. moellendorffii* genome, but we were unable to find any. miR390 was also not found in our small RNA-seq or in the genome of *S. moellendorffii*. miR168 was known to be present in the ancient euphyllophytes, since it was identified in the fern *M. quadrifolia* and in angiosperms [28]. As miR168 targets *AGO1* in angiosperms, we interrogated whether this miR-target relationship was established in ferns. We found that only in *E. ramosissimum* did a miR168 candidate (Era-miR168.921) potentially target Eram1374, an *AGO* in the *AGO1*/*5*/*10* clade (Additional file 1: Table S2 and S5). Interestingly, fern miRNA candidates from two other families, Ani-miR848.276 and Sun-miR159-5p.2545, could potentially target *AGO* homologs Anim17854 (in the *AGO2*/*3*/*7* clade) and Sunm18764 (in the *AGO1*/*5*/*10* clade) (Additional file 1: Table S5). Thus, the miR168-*AGO1* relationship in angiosperms was likely established after the divergence of ferns and seed plants.

### miRNA targets are conserved among vascular plants

**Fig. 8:**
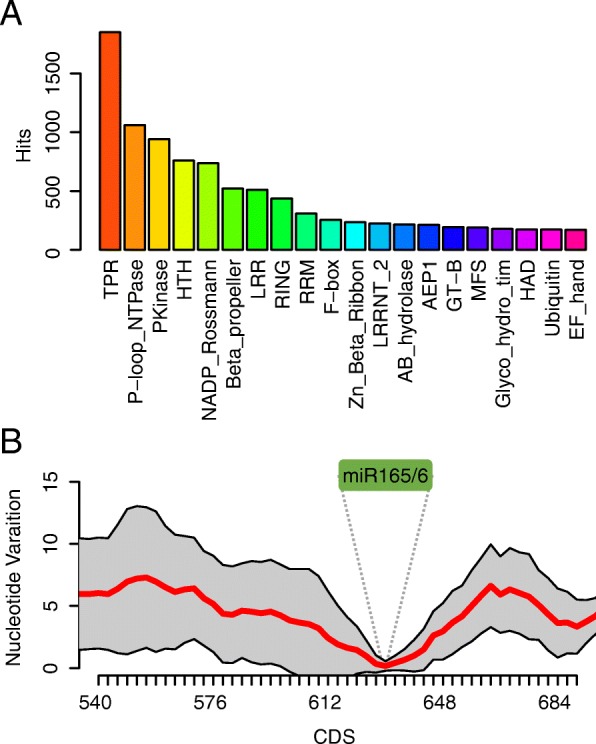
Predicted targets of miRNA candidates in lycophytes and ferns. **a** Top 20 protein superfamily or domain hits in predicted targets of miRNA candidates. Each color represents one type of protein family or domain. The *y-axis* shows the numbers of hits in domain searches using all predicted targets of miRNA candidates from lycophytes and ferns. **b** Nucleotide variations in a group of orthologous genes targeted by miR166 family members. The coding sequences of these genes were aligned using 21-nt sliding windows. The sequences were perfectly aligned at the miR166 binding sites. Nucleotide variations within each window were calculated and plotted. The *x-axis* positions represent those of the 5′ ends of windows. The *red line* indicates the average nucleotide variations at each position and the *grey area* shows the standard errors of averages. The *green* “*miR166*” label and *dotted lines* mark the location of the binding sites of miR166 family members

miRNAs function by repressing target genes; thus, finding their targets in corresponding samples could elucidate the biological roles of the predicated miRNAs in a functional context. To achieve this purpose, we performed miRNA target prediction. We found that 48% of the miRNA candidates had predicted target mRNAs in the same samples from which the miRNAs were detected (Table 1). In angiosperms, a number of gene families encoding known protein domains have been reported as miRNA targets [72] (Fig. 8a), such as *PPR* (*TPR* superfamily), *F-box* and *SBP* genes, etc. By searching for protein domains in the predicted miRNA targets in lycophytes and ferns, we found that the miRNA–target pairs were similar to those in angiosperms (Fig. 8a). For example, the miR172 family targets AP2 family genes involved in many developmental processes such as flowering and floral development [66]. Our analyses of lycophyte and fern datasets predicted 28 miR172-3p and 10 miR172-5p in 20 samples and found 104 putative targets, including 45 *AP2* family members (Additional file 1: Table S5). This greatly expands previous findings of conservation in miRNA–target pairs based on studies with a few non-angiosperm plants [72]. In addition, we found an orthologous group of genes targeted by the conserved miR166 family members. The miRNA-binding sites in these genes were significantly more conserved compared to flanking sequences (Fig. 8b), suggesting higher selection pressure on these miRNA-binding sites. These results not only support our accurate prediction of miRNA–target pairs but also agree with the co-evolution between miRNAs and their targets [73]. To verify the predicted miRNA–target relationship, we performed degradome/PARE sequencing [74] of *S. moellendorffii* to identify cleaved RNA fragments. Nine peaks were most likely generated by annotated miRNAs or miRNA candidates as predicted in this study, and these miRNAs included the highly abundant miR156/7 and miR165/6 that were detected in our small RNA sequencing (Additional file 2: Figure S8; Additional file 1: Table S3; Additional file 1: Table S6). Some miRNA candidates such as Smo-miR156/7-5p.2350 were not reported before, but we confirmed their existence in the genome and their function in mRNA cleavage (Additional file 2: Figure S8b). Strikingly, Smo-miR319-3p, which could not be mapped to the genome, was found to match the cleavage of the transcript Smo10655 (Additional file 2: Figure S8c), strongly supporting Smo-miR319-3p as an authentic miRNA and implying the incompleteness of genome sequencing.

**Table 1:** Information on miRNA candidates and target transcripts in various species

Species	Number of miRNA candidates	Number of predicted targets	Number of miRNA candidates with targets
*Lycopodium cernuum*	51	154	31
*Selaginella uncinata*	98	308	72
*Selaginella moellendorffii*	209	653	162
*Isoetes sinensis*	121	245	87
*Equisetum ramosissimum*	81	182	49
*Psilotum nudum*	79	188	49
*Ophioglossum vulgatum*	73	227	46
*Angiopteris fokiensis*	126	230	66
*Osmunda vachellii*	133	429	100
*Vandenboschia striata*	67	165	46
*Dicranopteris pedata*	199	246	101
*Diplopterygium chinense*	234	280	159
*Lygodium japonicum*	73	142	35
*Salvinia cucullata*	155	396	108
*Salvinia molesta*	36	278	34
*Azolla caroliniana*	90	186	58
*Cibotium barometz*	85	246	44
*Alsophila spinulosa*	112	224	63
*Sphenomeris chinensis*	97	667	60
*Pteris vittata*	119	490	84
*Microlepia platyphylla*	69	140	52
*Asplenium nidus*	62	296	49
*Diplazium esculentum*	132	194	71
*Cyrtomium fortunei*	97	351	74
*Phymatosorus cuspidatus*	77	239	39

## Discussion

Small RNAs constitute an important population of the RNA complement in eukaryotes. Our current knowledge of small RNAs has been derived mainly from model plants with sequenced genomes, most of which are angiosperms. In angiosperms, most 21-nt small RNAs are miRNAs, and most 24-nt small RNAs are generated by Pol IV and act in the RdDM pathway. Sister species of seed plants, including lycophytes and ferns, are underrepresented in the small RNA field due to the lag in genome sequencing. This has precluded a holistic picture of small RNA pathways in land plants. Our characterization of small RNAs and identification of small RNA pathway genes in lycophytes and ferns, two major sister lineages of seed plants, filled a major gap in our knowledge of small RNAs. The inclusion of all major lycophyte and fern lineages in phylogenetic analyses of small RNA pathway genes, which was not previously possible, led to new insights into small RNA pathway evolution in land plants.

### Conservation and diversification of small RNA pathways in land plants

Although RNA-seq-based gene identification may miss some genes due to their spatiotemporally restricted expression, the inclusion of multiple species in each phylogenetic group helped to minimize the influence of incomplete sampling of tissues. As a result, homologs of angiosperm genes in small RNA pathways were identified from most species of lycophytes and ferns. Homologs of these genes were also found in the sequenced genomes of two byrophytes and three algae. Thus, it can be concluded that small RNA-based RNA silencing was established in the common ancestor of green plants. Our phylogenetic analyses reveal that small RNA pathways diversified and specialized in the most recent common ancestor of land plants. For example, all angiosperms have at least four classes of *DCLs* that generate different types of small RNAs (miRNAs and various endogenous and viral siRNAs). Each *DCL* class is a monophyletic group in land plants (Additional file 2: Figure S1), indicating ancient divergence of the *DCL* family and implying ancient diversification of small RNA pathways in land plants. *DCL2* genes were not detected in lycophytes and some ferns, and was absent in the genome of the bryophyte *P. patens*, suggesting that *DCL2* was lost in some bryophytes, lycophytes, and some ferns or it evolved after the divergence between lycophytes and other vascular plants.

Consistent with the early diversification of *DCL*s, the *AGO* family also diversified early in land plants, as all three major clades of angiosperm *AGOs* have counterparts in bryophytes. As different AGO proteins bind to and mediate the activities of different classes of small RNAs, the early diversification of the *AGO* family supports ancient diversification of small RNA pathways in land plants. We also discovered differences in *AGO* evolution compared to that of *DCL* genes. The *AGO* genes of vascular plants form several separate groups (Additional file 2: Figure S2) in each of three conserved clades. This indicates that the expansion of *AGO* genes in each of the three clades occurred in parallel in seed plants and their sister species. For example, specific *AGO* genes in angiosperms, such as *AthAGO10*, which sequesters miR166 from AGO1 in *Arabidopsis* [75], and *OsAGO18*, which functions in miRNA sequestration in rice [76], emerged after the divergence of ferns and seed plants. Thus, the functions of these genes in *Arabidopsis* and rice do not apply to ferns and lycophytes. In addition, the expansion of the *AGO1*/*5*/*10* and *AGO2*/*3*/*7* clades in sister species of seed plants is limited to leptosporangiates, the most prosperous lineage of ferns, including Polypodiales, Salvinales, and other orders (Additional file 2: Figure S2a, b). This observation raised the possibility that *AGO* gene expansion and functional divergence of small RNA pathways could have contributed to plant survival.

Our studies also shed light on the evolution of RdDM in land plants. Although DNA methylation has been described in the moss *P. patens* and the lycophyte *S. moellendorffii*, evidence for the existence of RdDM in other lycophytes was lacking [54, 77]. Here we identified many key genes involved in RdDM not only in ferns but also in lycophytes, including subunits specific to Pol IV/Pol V. This implies a developed RdDM pathway in lycophytes as in ferns. However, mirroring previous findings, we did not find abundant 24-nt small RNAs in many species, including two lycophytes and ten ferns. Most of the ferns possessing a 24-nt small RNA peak are in Polypodiales, the most diversified order among extant ferns (Fig. 4a; Additional file 2: Figure S6). The earth underwent a vegetation change from a landscape dominated by gymnosperms and seed-free vascular plants to one populated mainly by angiosperms [78]. In this change most ancient species were compromised when the angiosperms began to bloom [79], while Polypodiales was an exception, which expanded as flowering plants flourished according to fossil and living records [80], and accounts for more than 80% of all extant fern species [80, 81]. The existence of 24-nt small RNAs and the expansion of AGOs in Polypodiales implied the emergence of a sophisticated system of generating and utilizing 24-nt small RNAs. We spectulate that the well developed regulatory system might have helped plants adapt to environments to facilitate survival [4] in two successful lineages of land plants, Polypodiales and angiosperms. It is possible that once the AGO family expanded to develop the capability of binding the 24-nt small RNAs, this class of small RNAs began to be predominant in the small RNA population and became vital in gene and genome regulation.

Our phylogenetic analyses show that ferns, but not lycophytes or bryophytes, have clear *NRPD1* and *NRPE1* genes. We prospose a model of the evolution of Pol IV and Pol V (Fig. 4d). An *NRPD1*/*E1*-like gene diverged from *NRPB1* in ancient land plants. As two *P. patens NRPD1*/*E1*-like genes have an *NRPE1*-like domain configuration (Fig. 3), the ancestral *NRPD1*/*E1*-like gene might have been more like *NRPE1*, but this is largely speculative. In ferns and angiosperms, *NRPD1* and *NRPE1* evolved from the *NRPD1*/*E1*-like ancestral gene. *NRPE1* in ferns lost the DeCL domain while *NRPE1* in angiosperms retained the DeCL domain. Notably, the role of *NRPD1* in generating 24-nt siRNAs in angiosperms may be acquired later in evolution, as in lycophytes and ferns, a correlation between 24-nt siRNAs and *NRPE1* expression was found (Fig. 4c).

### Insights into miRNA evolution in land plants

The molecular framework of miRNA biogenesis, degradation, and mode of action was likely established in the common ancestor of land plants, as major components of the miRNA pathway as we understand in angiosperms have homologs in bryophytes, lycophytes, and ferns, including DCL1 and SE in miRNA biogenesis, HEN1 in miRNA stability, AGO1 in miRNA binding and activity, and SDN, HESO1, and URT1 in miRNA turnover. We found many truncated and tailed isoforms in miRNA candidates, with the miR165/6 family from *S. moellendorffii* being a prominent example (Fig. 6b). This suggests that the mechanisms of miRNA degradation are conserved in land plants. Degradome/PARE analysis of *S. moellendorffii* not only confirmed our miRNA–target predictions but also demonstrated that miRNA-guided target RNA cleavage occurs in a lycophyte.

Our identification of conserved miRNAs in lycophytes and ferns, together with previous studies of miRNAs in two ferns, *M. quadrifolia* and *P. minima* [28, 30], has enriched our understanding of the evolution of miRNAs. These studies point to approximately 12 miRNA families that were present in the most recent common ancestor of vascular plants by adding more lycophytes into the species pool. In addition, our study helped time the origination of a few well-known, conserved miRNAs. We found that some well-known, conserved miRNAs were missing in lycophytes, including miR172, miR390, miR160, miR530, miR168, miR394, and miR169 (Fig. 7). Since miR172 was not found in the moss *P. patens* [29] or the liverworts *Pellia endiviifolia* [82] and *M. polymorpha* [38], it is probable that miR172 evolved only in the most recent common ancestor of ferns and seed plants to regulate *AP2* genes. Many miR172-*AP2* pairs were found in ferns, but no *AP2* genes were among the predicted miRNA targets in *S. moellendorffii* and other lycophytes, except for a miR171-3p-*AP2* pair in *L. cernuum* (Additional file 1: Table S5). This implies that lycophyte *AP2* genes are not regulated by miR172 or any miRNA except for *L. cernuum*, in which they are regulated by miR171-5p.1360. Also, as a trigger for phasiRNA biogenesis, miR390 was considered conserved in land plants due to the presence of this miRNA and its target *TAS3* in the moss *P. patens* [71]. In our study, no miR390 candidates were found in any of the lycophytes, raising the possibility that the phasiRNA pathway was not established in lycophytes. The absence of several other phasiRNA-triggering miRNAs in lycophytes as well as the absence of *PHAS* or *TAS* genes or phased siRNAs in *S. moellendorffii* support this hypothesis. Further genome information of these species would help evaluate the existence/absence of secondary siRNAs in lycophytes.

This work also revealed fluid miRNA–target relationships in evolution. While *AGO1* is conserved in land plants, miR168, which targets *AGO1* in angiosperms, probably evolved in the most recent common ancestor of vascular plants [28]. In some lycophytes, miRNAs other than miR168 target *AGO1* (Additional file 1: Table S5). Thus, different miRNAs are employed to target *AGO1* in different lineages. Another fluid relationship is AGO–miRNA association. In angiosperms, AGO10 specifically associates with miR165/6 and sequesters it from AGO1 [75]. While the miR165/6 family is conserved in land plants, AGO10 only emerged in angiosperms after the fern–seed plant divergence. Thus, the specific AGO10-miR165/6 relationship in angiosperms evolved after this divergence.

## Conclusions

This study on lycophytes and ferns, two major land plant lineages that are extremely underrepresented in small RNA studies, sheds light on the evolution of small RNA pathways and miRNAs in land plants and provides valuable information from sister lineages of seed plants for comparative studies. Three DCL and four AGO clades formed early in land plants, implicating functional diversification of small RNAs early in land plant evolution. The molecular framework for miRNA biogenesis, degradation, and activity was likely established in the common ancestor of land plants. The lack of certain subunits of Pol IV/Pol V together with the absence of prominent 24-nt siRNAs in lycophytes and some ferns suggests stepwise assembly of the RdDM machinery in land plant evolution. The existence of an AGO clade in bryophytes, lycophytes, ferns, and possibly gymnosperms but not angiosperms strongly suggests differences in RNA silencing between angiosperms and other land plants. Our analyses of miRNAs have enriched the landscape of conserved miRNA families in major land plant lineages. The landscape of miRNAs in sister species of seed plants also has ramifications on the origin of phasiRNAs.

## Methods

### Plant sample collection and total RNA extraction

A total of four lycophytes and 21 fern species were selected following these rules: 1) at least one representative in each of the three and 11 current orders of lycophytes and ferns, respectively; 2) more species in family-rich orders; 3) priority given to ones with sequenced genomes. Other information about the selected species is detailed in Additional file 1: Table S1. Among these species, only *S. moellendorffii* has a published whole-genome sequence [83]. For most samples, young aerial parts were collected and total RNA was extracted from ground plant tissues using a modified CTAB method as described by Zhang et al. [84].

### mRNA library construction, sequencing, and data processing

RNA-seq libraries were constructed using TruSeq RNA Library Prep Kit v2 (Illumina, RS-122-2001 and RS-122-2002), and pooled and sequenced (paired-end, 125 bp) on the Illumina Hiseq 2500 platform at Berry Genomics (Beijing, China) or on the Illumina Hiseq 3000 platform (paired-end, 150 bp) at Genergy (Shanghai, China). All sequences were used for *de novo* transcript assembly using Trinity v2.1.1 [85] with parameters “--trimmomatic --quality_trimming_params ‘ILLUMINACLIP:/usr/local/bin/trinity-plugins/Trimmomatic/adapters/TruSeq3-PE.fa:2:30:10 SLIDINGWINDOW:4:20 LEADING:10 TRAILING:10 MINLEN:70’ --normalize_max_read_cov 100 --min_kmer_cov 2”. Assembled transcripts over 100 nt were named by a combination of species abbreviation, “m” meaning “mRNA”, and a uniform serial number, such as Pcum53368. Afterwards, proteins were predicted from the assembled transcripts using TransDecoder with default parameters, which is integrated in Trinity, and used for protein domain search with hmmsearch [86].

### Phylogenetic analysis of proteins related to small RNA pathways

Proteins that are putative components of small RNA pathways were identified through hmmsearch using an HMM profile built from their homologs in *A. thaliana*, *O. sativa*, *A. trichopoda*, *S. moellendorffii*, and *P. patens* obtained from Phytozome v12 (https://phytozome.jgi.doe.gov). The identified protein sequences were aligned by MUSCLE v3.8.31 with default parameters [87] and optimized manually. The corresponding coding nucleotide sequences (CDS) from the protein alignment were extracted and aligned, and the nucleotide alignment was used for subsequent phylogenetic analyses. The maximum likelihood (ML) trees were inferred using RAxML v8.2.4 [88] with the parameter “-f a -m GTRCAT -N 1000” and drawn by MEGA7 [89]. Genes in each tree were labeled with the species names, and their nucleotide sequences are included in Additional file 3.

### Small RNA library construction, sequencing, and data processing

Total RNA (20 μg) from each sample was resolved in a 15% polyacryladmide/urea gel. Gel slices corresponding to RNA sizes of 15–40 nt were excised, and RNA was extracted using the TRIzol@ reagent (ThermoFisher, 15596026). The RNA was then used to construct small RNA libraries according to instructions from NEBNext® Multiplex Small RNA Library Prep Set for Illumina® (NEB, E7300S). The small RNA libraries were then pooled and sequenced (single-end reads of 50 bp) on the Illumina HiSeq 2500 platform at Berry Genomics (Beijing, China). The adapter sequence was removed from the output reads using cutadapt v1.10 [90], and only 16–26-nt-long reads were retained. The rRNA, tRNA, snoRNA, and snRNA fragments were filtered against the *S. moellendorffii* genome with bowtie v1.1.2 [91] allowing for two mismatches and removed. Any 3′ U tails were shortened to 1 nt to minimize the influence on subsequent analyses. The prediction of conserved miRNAs was conducted following the method described in [28]. Only those with less than 3-nt mismatches and less than 2-nt differences in length were recognized as miRNA candidates and assigned as the closest homologs to the reported plant miRNAs. In this way, similar miRNA families could be combined into one family, such as miR156/157, miR165/166, and miR170/171. Predicted miRNAs with identical 5′ nucleotides 1–16 were combined into one miRNA cluster with the most abundant one as the representative (Additional file 2: Figure S8), and labeled with a uniform serial number followed by the raw read counts, such as Smo-miR170/1-3p.2380_3761. The abundance of these miRNA candidates was calculated as reads per million (RPM) total reads of 18–26 nt small RNAs in each species. The miRNA candidates with more than ten raw reads or five in RPM (whichever is higher) were retained. All identified miRNA candidates are included in Additional file 4.

### Degradome/PARE library construction, sequencing, and data processing

The *S. moellendorffii* degradome/PARE library was constructed with 75 μg of total RNA as described [74]. The library was sequenced on the Illumina HiSeq 2500 platform (single end, 50 bp). After removing adaptor sequences, reads shorter than 19 nt were removed. Retained reads were then mapped to the transcripts assembled from mRNA-seq using ShortStack with default parameters. A local algorithm with requirements for peak calling was employed: 1) at least 12 unique tags were mapped to the transcript; 2) the peak position is among the top 12 positions with mapped reads in this transcript; 3) the RPM of the peak is over 5; 4) the RPM of the peak is larger than the mean plus five times standard error of RPM of all positions with mapped reads on this transcript.

### Genome mapping of small RNAs in *S. moellendorffii*

The small RNA reads in *S. moellendorffii* were mapped to the genome using ShortStack [92] allowing for no mismatches. The 400-bp flanking sequences of aligned hits were extracted and subjected to secondary structure prediction using RNAfold in ViennaRNA packages 2 [93] with default parameters. The miRNA candidates with less than four mismatches in the hairpin structures were retained.

### miRNA target analysis

The prediction of miRNA targets in each species was performed using TarHunter (https://github.com/XMaBio/TarHunter) with a cutoff of 2.5. Predicted targets were searched for known protein domains using hmmsearch with the Pfam-A.hmm file downloaded from Pfam (http://pfam.xfam.org). Orthologous targets in all species were also predicted by TarHunter, and orthologous groups present in over 15 species were retained for analysis of sequence variations. Aligned nucleotide sequences within a group were divided into 30-nt sliding windows to calculate nucleotide variation as compared to the ancestral sequence calculated by MEGA7.

## Additional files

Additional file 1: Supplemental tables. (XLSX 758 kb)

Additional file 2: Supplemental figures and legends. (PDF 2938 kb)

Additional file 3: Nucleotide sequences of genes identified in this study. (TXT 3511 kb)

Additional file 4: Conserved miRNAs identified in this study. (TXT 112 kb)
