# Supplementary material for: Conservation and divergence of small RNA pathways and microRNAs in land plants
Source: Genome Biol. 2017 Aug 23;18:158. doi: 10.1186/s13059-017-1291-2 (PMC5569507; doi:10.1186/s13059-017-1291-2)
Supplement: Supplementary file 2 — Supplemental figures and legends. (PDF 2938 kb) [file 13059_2017_1291_MOESM2_ESM.pdf]

Figure S1

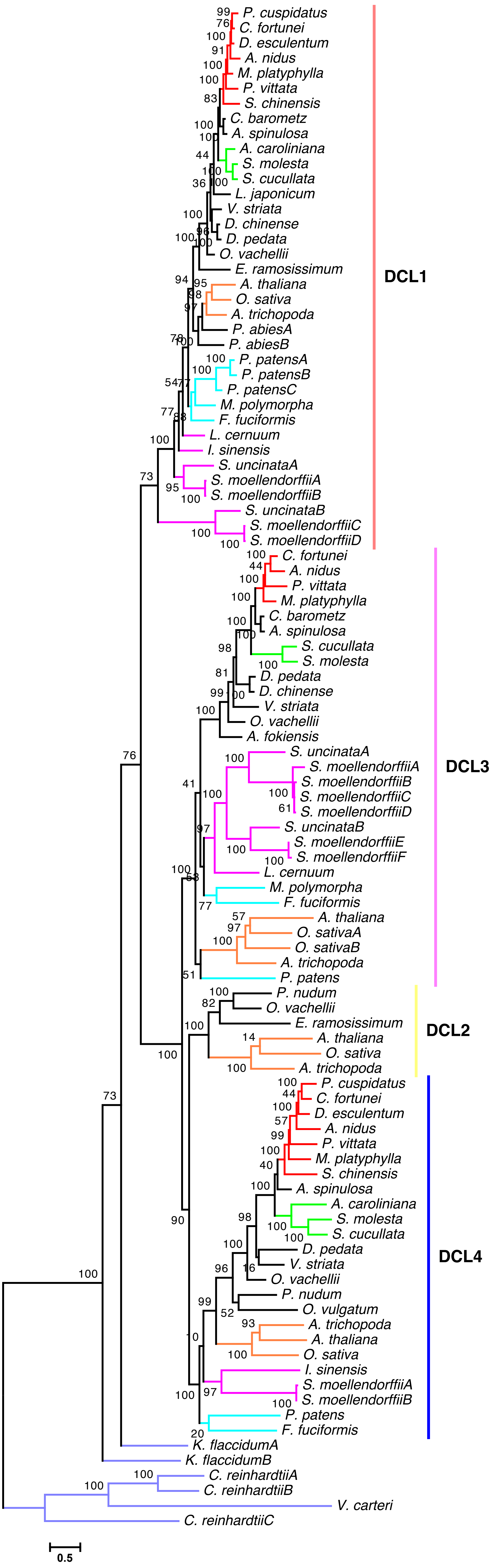
P. cuspidatus
C. fortunei
D. esculentum
A. nidus
M. platyphylla
P. vittata
S. chinensis
C. barometz
A. spinulosa
A. caroliniana
S. molesta
S. cucullata
L. japonicum
V. striata
D. chinense
D. pedata
O. vachellii
E. ramosissimum
A. thaliana
O. sativa
A. trichopoda
P. abiesA
P. abiesB
P. patensA
P. patensB
P. patensC
M. polymorpha
F. fuciformis
L. cernuum
I. sinensis
S. uncinataA
S. moellendorffiiA
S. moellendorffiiB
S. uncinataB
S. moellendorffiiC
S. moellendorffiiD
DCL1
C. fortunei
A. nidus
P. vittata
M. platyphylla
C. barometz
A. spinulosa
S. cucullata
S. molesta
D. pedata
D. chinense
V. striata
O. vachellii
A. fokiensis
S. uncinataA
S. moellendorffiiA
S. moellendorffiiB
S. moellendorffiiC
S. moellendorffiiD
S. uncinataB
S. moellendorffiiE
S. moellendorffiiF
L. cernuum
M. polymorpha
F. fuciformis
A. thaliana
O. sativaA
O. sativaB
A. trichopoda
P. patens
DCL3
P. nudum
O. vachellii
E. ramosissimum
A. thaliana
O. sativa
A. trichopoda
DCL2
P. cuspidatus
C. fortunei
D. esculentum
A. nidus
P. vittata
M. platyphylla
S. chinensis
A. spinulosa
A. caroliniana
S. molesta
S. cucullata
D. pedata
V. striata
O. vachellii
P. nudum
O. vulgatum
A. trichopoda
A. thaliana
O. sativa
I. sinensis
S. moellendorffiiA
S. moellendorffiiB
P. patens
F. fuciformis
DCL4
K. flaccidumA
K. flaccidumB
C. reinhardtiiA
C. reinhardtiiB
V. carteri
C. reinhardtiiC
0.5

Figure S1 An ML tree of *DCL* genes in bryophytes, lycophytes, ferns, and angiosperms. The nomenclature is based on the annotated *Arabidopsis* genes *DCL1*, *DCL2*, *DCL3* and *DCL4*. The detailed *DCL1* clade is shown in Figure 2A. Purple, green algae; Light blue, bryophytes; Magenta, lycophytes; Red, Polypodiales (ferns); Green, Salviniales (ferns); Orange, angiosperms; Black, other ferns and gymnosperm. The species and sequence accessions used in the analysis are shown in Additional file 1: Table S2. The scale bar represents nucleotide substitution rates. Numbers beside nodes are bootstrap support values indicating confidence (from 0 to 100). Alignment length: 22788 nt.

Figure S2

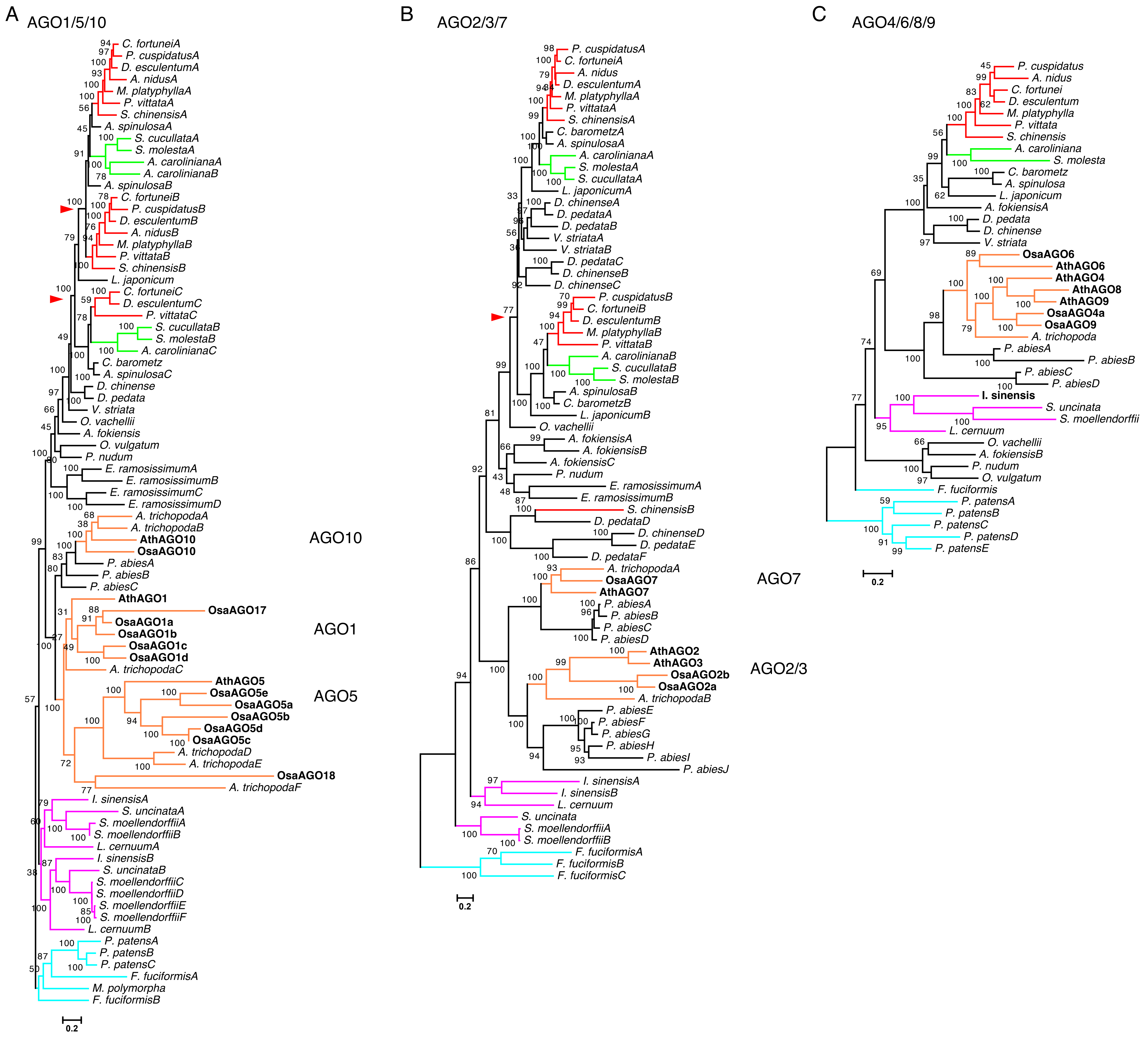

A
AGO1/5/10
C. fortuneiA
P. cuspidatusA
D. esculentumA
A. nidusA
M. platyphyllaA
P. vittataA
S. chinensisA
A. spinulosaA
S. cucullataA
S. molestaA
A. carolinianaA
A. carolinianaB
A. spinulosaB
C. fortuneiB
P. cuspidatusB
D. esculentumB
A. nidusB
M. platyphyllaB
P. vittataB
S. chinensisB
L. japonicum
C. fortuneiC
D. esculentumC
P. vittataC
S. cucullataB
S. molestaB
A. carolinianaC
C. barometz
A. spinulosaC
D. chinense
D. pedata
V. striata
O. vachellii
A. fokiensis
O. vulgatum
P. nudum
E. ramosissimumA
E. ramosissimumB
E. ramosissimumC
E. ramosissimumD
A. trichopodaA
A. trichopodaB
AthAGO10
OsaAGO10
P. abiesA
P. abiesB
P. abiesC
AthAGO1
OsaAGO17
OsaAGO1a
OsaAGO1b
OsaAGO1c
OsaAGO1d
A. trichopodaC
AthAGO5
OsaAGO5e
OsaAGO5a
OsaAGO5b
OsaAGO5d
OsaAGO5c
A. trichopodaD
A. trichopodaE
OsaAGO18
A. trichopodaF
I. sinensisA
S. uncinataA
S. moellendorffiiA
S. moellendorffiiB
L. cernuumA
I. sinensisB
S. uncinataB
S. moellendorffiiC
S. moellendorffiiD
S. moellendorffiiE
S. moellendorffiiF
L. cernuumB
P. patensA
P. patensB
P. patensC
F. fuciformisA
M. polymorpha
F. fuciformisB
AGO10
AGO1
AGO5
0.2
B
AGO2/3/7
P. cuspidatusA
C. fortuneiA
A. nidus
D. esculentumA
M. platyphyllaA
P. vittataA
S. chinensisA
C. barometzA
A. spinulosaA
A. carolinianaA
S. molestaA
S. cucullataA
L. japonicumA
D. chinenseA
D. pedataA
D. pedataB
V. striataA
V. striataB
D. pedataC
D. chinenseB
D. chinenseC
P. cuspidatusB
C. fortuneiB
D. esculentumB
M. platyphyllaB
P. vittataB
A. carolinianaB
S. cucullataB
S. molestaB
A. spinulosaB
C. barometzB
L. japonicumB
O. vachellii
A. fokiensisA
A. fokiensisB
A. fokiensisC
P. nudum
E. ramosissimumA
E. ramosissimumB
S. chinensisB
D. pedataD
D. chinenseD
D. pedataE
D. pedataF
A. trichopodaA
OsaAGO7
AthAGO7
P. abiesA
P. abiesB
P. abiesC
P. abiesD
AthAGO2
AthAGO3
OsaAGO2b
OsaAGO2a
A. trichopodaB
P. abiesE
P. abiesF
P. abiesG
P. abiesH
P. abiesI
P. abiesJ
I. sinensisA
I. sinensisB
L. cernuum
S. uncinata
S. moellendorffiiA
S. moellendorffiiB
F. fuciformisA
F. fuciformisB
F. fuciformisC
AGO7
AGO2/3
0.2
C
AGO4/6/8/9
P. cuspidatus
A. nidus
C. fortunei
D. esculentum
M. platyphylla
P. vittata
S. chinensis
A. caroliniana
S. molesta
C. barometz
A. spinulosa
L. japonicum
A. fokiensisA
D. pedata
D. chinense
V. striata
OsaAGO6
AthAGO6
AthAGO4
AthAGO8
AthAGO9
OsaAGO4a
OsaAGO9
A. trichopoda
P. abiesA
P. abiesB
P. abiesC
P. abiesD
I. sinensis
S. uncinata
S. moellendorffii
L. cernuum
O. vachellii
A. fokiensisB
P. nudum
O. vulgatum
F. fuciformis
P. patensA
P. patensB
P. patensC
P. patensD
P. patensE
0.2

Figure S2 ML trees of AGO homologs in bryophytes, lycophytes and ferns. **(A)** the AGO1/5/10 clade; **(B)** the AGO2/3/7 clade; **(C)** the AGO4/6/8/9 clade. The AGO-like clade is shown in detail in Figure 2C. Red arrowheads indicate gene duplications in leptosporangiate ferns. Light blue, bryophytes; Magenta, lycophytes; Red, Polypodiales (ferns); Green, Salviniales (ferns); Orange, angiosperms; Black, other ferns and gymnosperm. The species names and sequence accessions are shown in Supplemental Table 2. The scale bar represents the nucleotide substitution rates. Numbers beside nodes are bootstrap support values showing confidence (from 0 to 100). Alignment length: AGO1/5/10, 6936 nt; AGO2/3/7, 5262 nt; AGO4/6/8/9, 3210 nt.

Figure S3

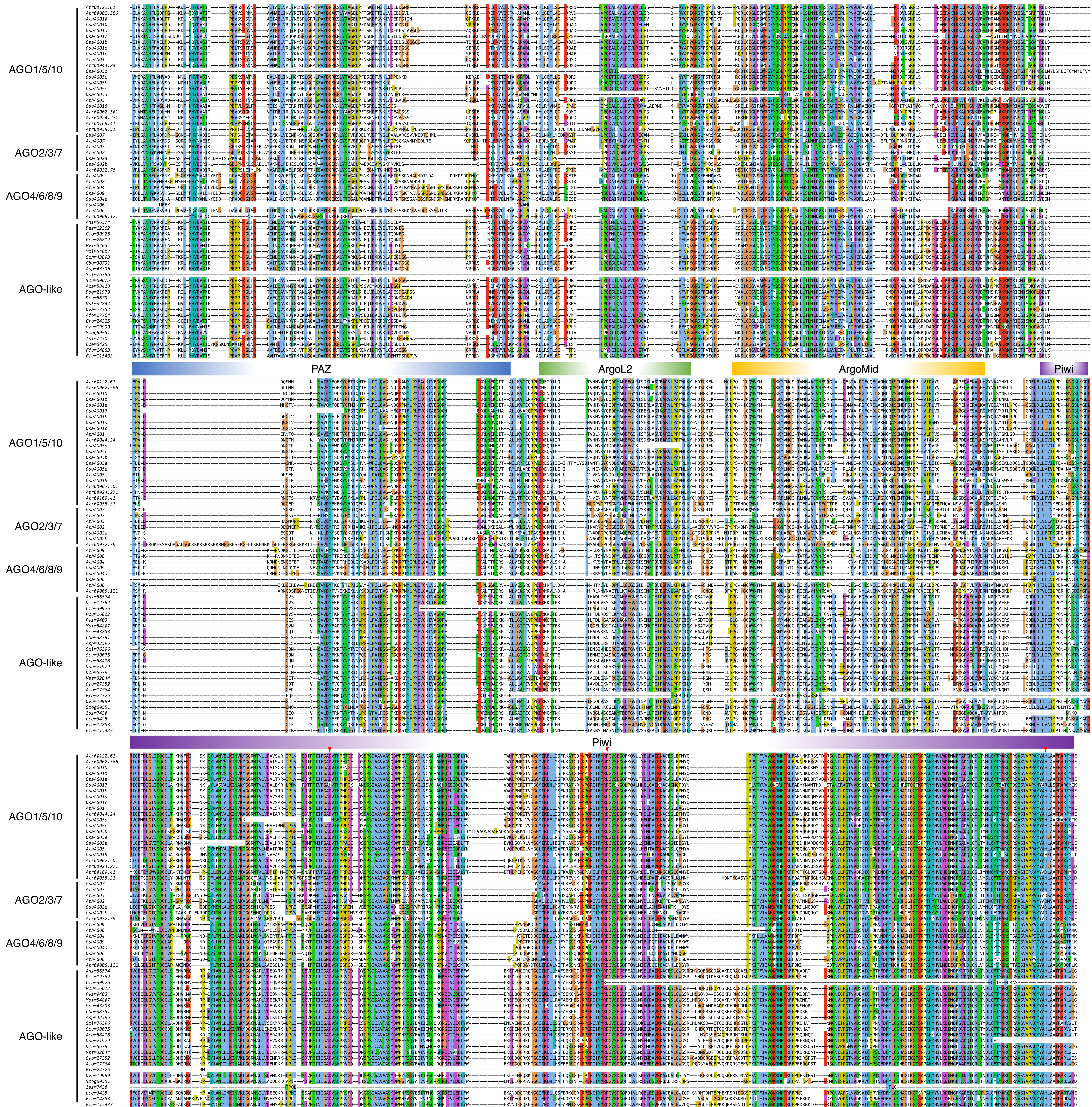

ArgoN
ArgoL1
PAZ
ArgoL2
ArgoMid
Piwi
AGO1/5/10
AGO2/3/7
AGO4/6/8/9
AGO-like

Figure S3 Amino acid sequence alignments of the conserved domains of representative AGO proteins. Amino acid sequences of angiosperm AGO1/5/10, AGO2/3/7 and AGO4/6/8/9 proteins were aligned with AGO proteins specific to the sister species of angiosperms. Sequences at the extreme N and C terminal ends are not shown. Protein domains identified by HMM are labeled at the top of the aligned sequences. Conserved catalytic DDH/D residues are labeled with red arrowheads.

Figure S4

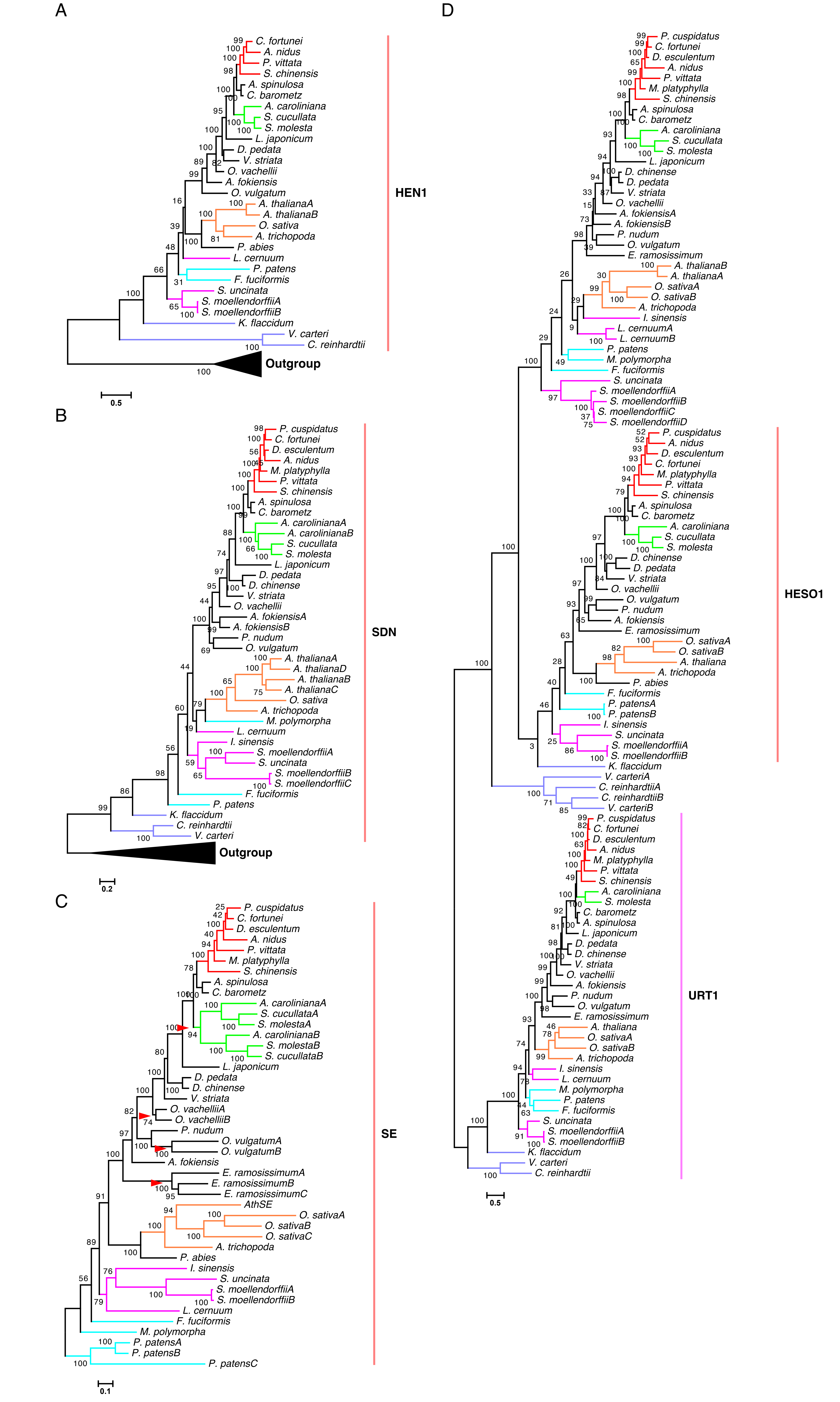

Figure S4 ML trees of various genes in miRNA biogenesis or degradation. **(A)** HEN1 with a clade of methyltransferases, which are homologs of the *Arabidopsis* gene AT4G25080, as the outgroup; **(B)** HESO1 and URT1 with a clade of nucleotidyltransferases, which are homologs of the *Arabidopsis* gene AT3G45750, as the outgroup; **(C)** SDN with a clade of polynucleotidyl transferases, which are homologs of *Arabidopsis* AT3G15080, as the outgroup; **(D)** SE with two algae genes, which contain the same protein domains, as the outgroup. Red arrowheads indicate gene duplication events. Purple, green algae; Light blue, bryophytes; Magenta, lycophytes; Red, Polypodiales (ferns); Green, Salviniales (ferns); Orange, angiosperms; Black, other ferns and gymnosperm. The species names and sequence accessions are shown in Additional file 1: Table S2. The scale bar represents the nucleotide substitution rates. Numbers beside nodes are bootstrap support values showing confidence (from 0 to 100). Alignment length: HEN1, 6333 nt; HESO1 and URT1, 7224 nt; SDN, 3903 nt; SE, 3312 nt.

Figure S5

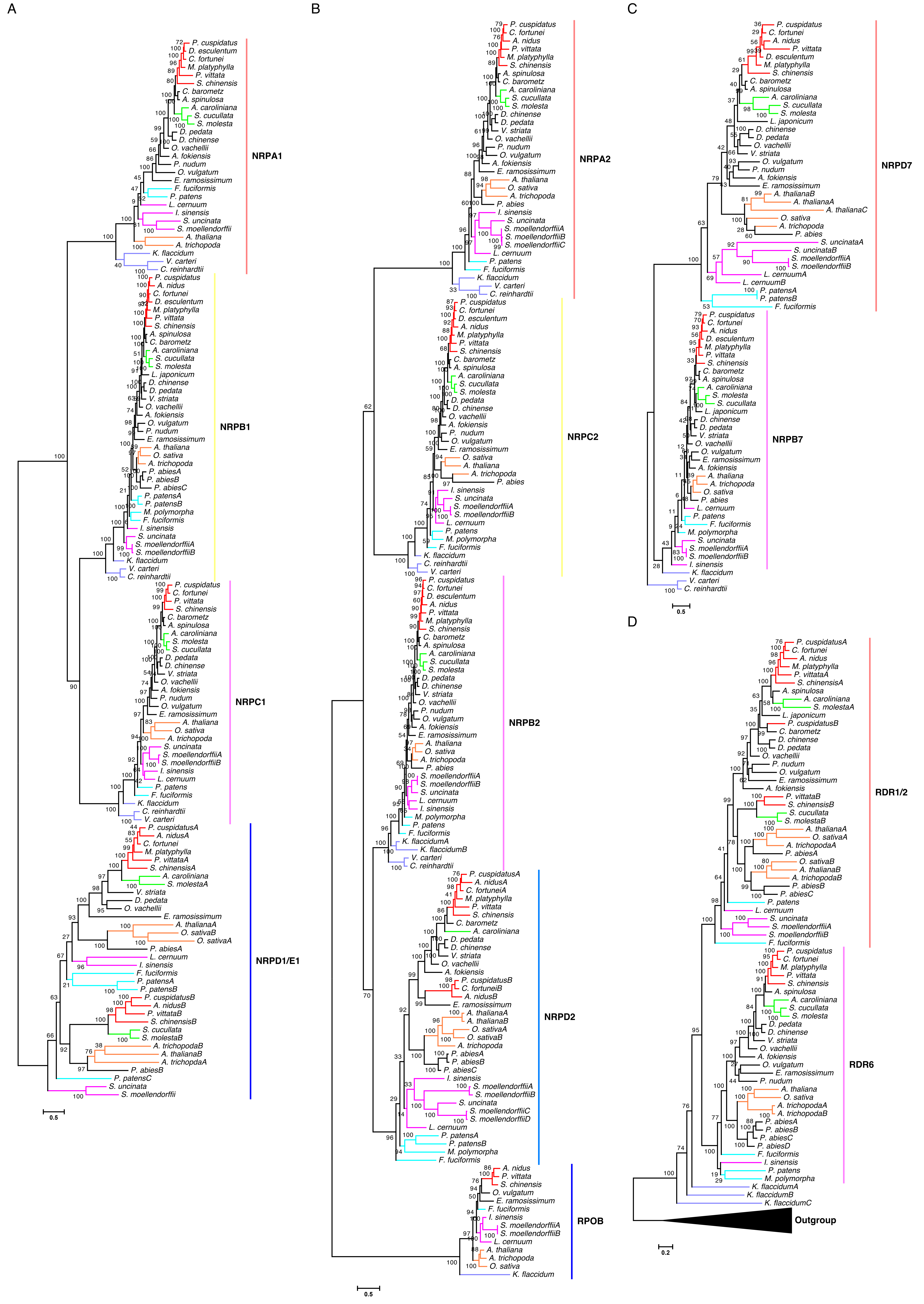
A
B
C
D
NRPA1
NRPB1
NRPC1
NRPD1/E1
NRPA2
NRPC2
NRPB2
NRPD2
RPOB
NRPD7
NRPB7
RDR1/2
RDR6
Outgroup
0.5
0.5
0.5
0.2

Figure S5 Phylogeny of genes involved in the RdDM pathway. ML trees were constructed with lycophyte, bryophyte, and fern homologs of specific subunits of angiosperm RNA Polymerases IV and V. To confidently assign homologs to Pol IV/Pol V relative to other RNA polymerases, homologs to all RNA polymerase subunits were included in the phylogenetic analyses. **(A)** NRPA1/B1/C1/D1/E1; **(B)** NRPB7/D7; **(C)** NRPA2/B2/C2/D2 with homologs of Arabidopsis Chloroplast RPOB as the outgroup; **(D)** RDR2/6 with homologs of Arabidopsis RDR4/5 as the outgroup. Purple, green algae; Light blue, bryophytes; Magenta, lycophytes; Red, Polypodiales (ferns); Green, Salviniales (ferns); Orange, angiosperms; Black, other ferns and gymnosperm. The species names and sequence accessions are shown in Additional file 1: Table S2. The scale bar represents the nucleotide substitution rates. Numbers beside nodes are bootstrap support values showing confidence (from 0 to 100). Alignment length: NRPA1/B1/C1/D1/E1, 10074 nt; NRPB7/D7, 1470 nt; NRPA2/B2/C2/D2, 7251 nt; RDR2/6, 6438 nt.

Figure S6

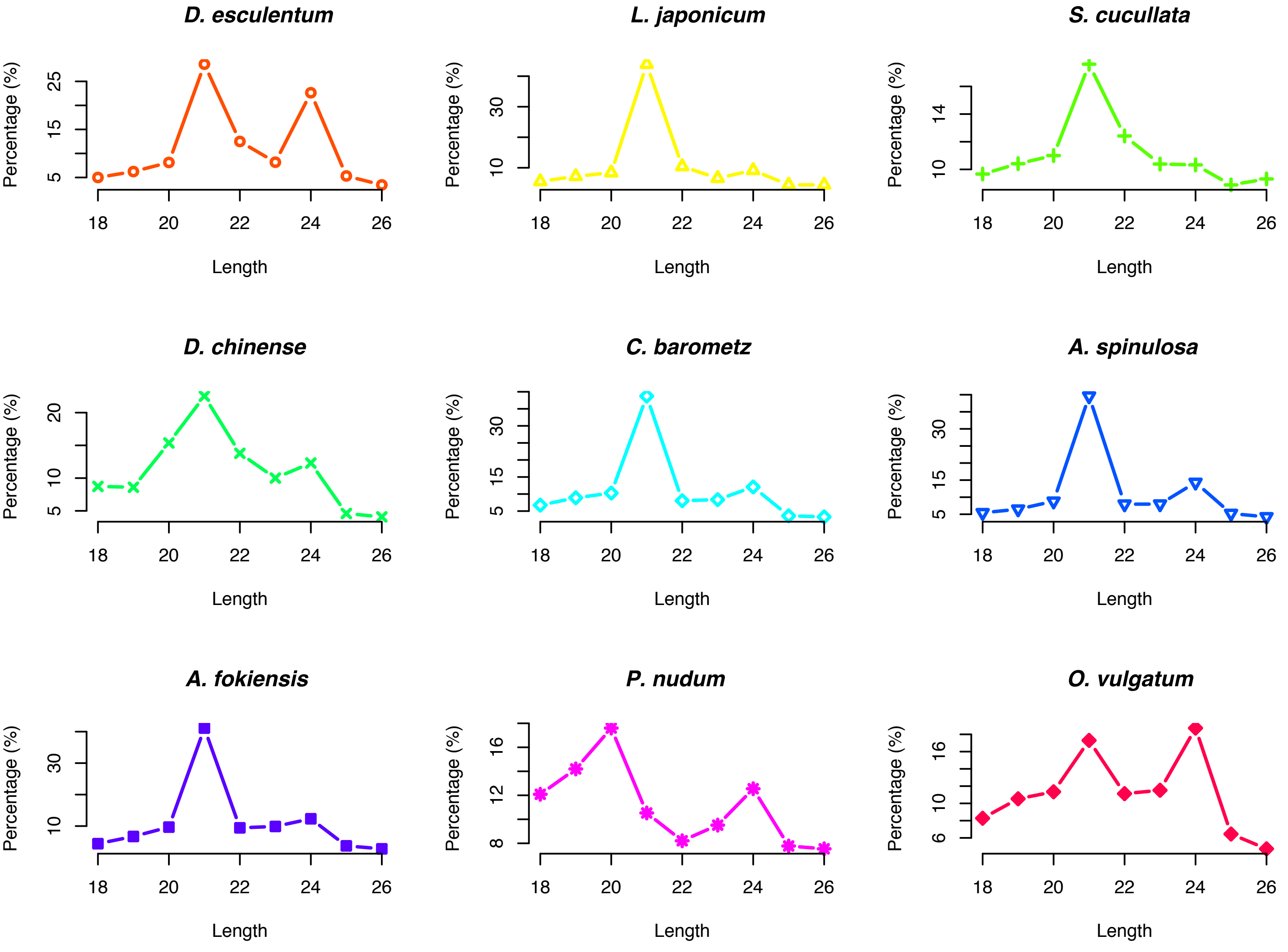

D. esculentum
L. japonicum
S. cucullata
D. chinense
C. barometz
A. spinulosa
A. fokiensis
P. nudum
O. vulgatum
Percentage (%)
Length
18
20
22
24
26

Figure S6 Size distribution of small RNA reads in various species. Relative proportions of small RNA reads as percentages of each size category in total reads. 9 ferns that are not shown in Figure 4A-B are shown here.

Figure S7

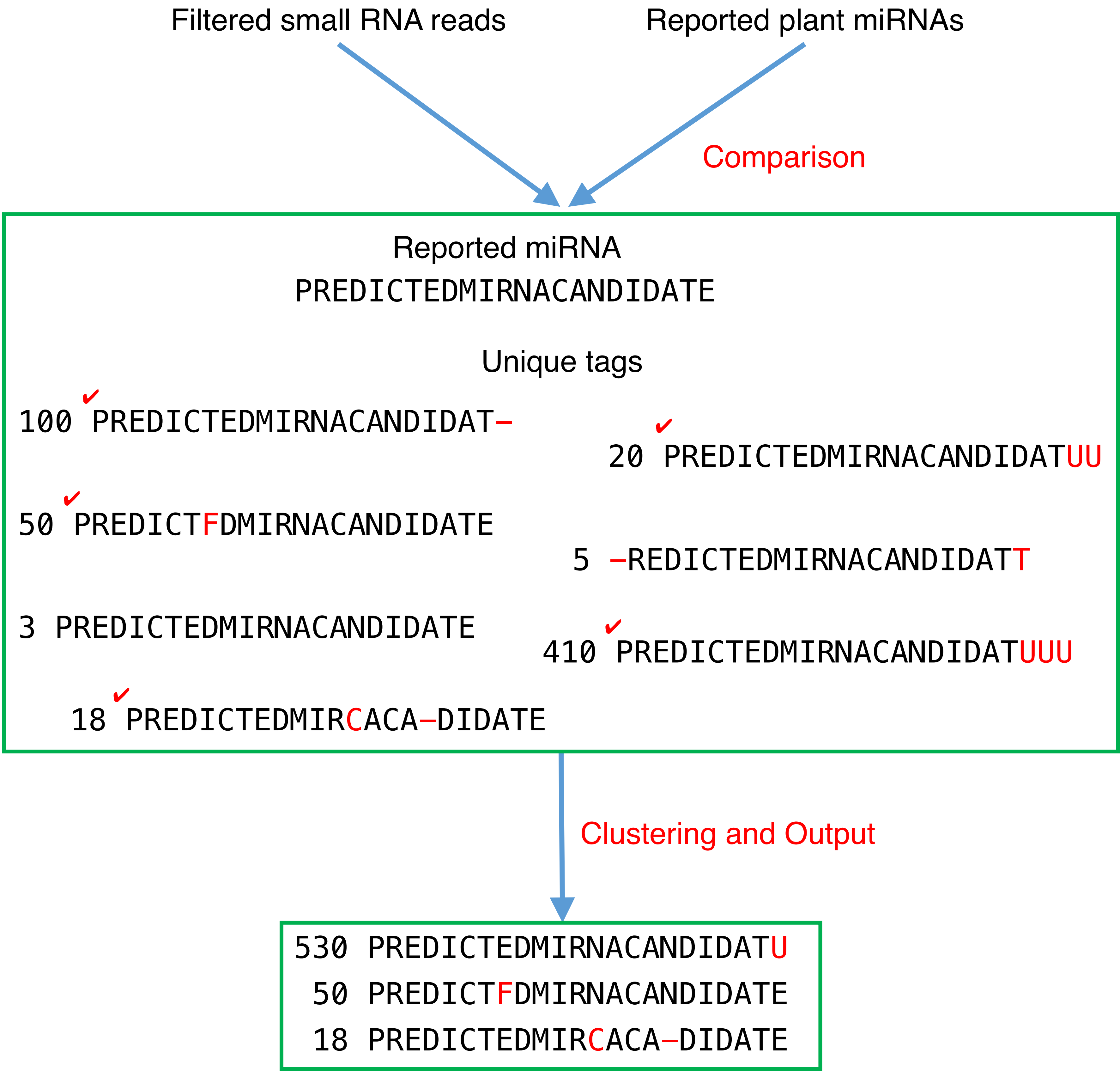

Filtered small RNA reads
Reported plant miRNAs
Comparison
Reported miRNA
PREDICTEDMIRNACANDIDATE
Unique tags
100 PREDICTEDMIRNACANDIDAT–
20 PREDICTEDMIRNACANDIDATUU
50 PREDICTFDMIRNACANDIDATE
5 –REDICTEDMIRNACANDIDATT
3 PREDICTEDMIRNACANDIDATE
410 PREDICTEDMIRNACANDIDATUUU
18 PREDICTEDMIRCACA–DIDATE
Clustering and Output
530 PREDICTEDMIRNACANDIDATU
50 PREDICTFDMIRNACANDIDATE
18 PREDICTEDMIRCACA–DIDATE

Figure S7 Schematic workflow for the prediction of conserved miRNAs. Small RNA reads after filtering are used to compare with annotated plant miRNAs in miRBase v21. Multiple-U tails of small RNAs are shortened to one single U. Unique tags with less than 3 mismatches, 2-nt differences in length, and at least 10 raw reads or 5 RPM (whichever is higher) are retained. Then unique tags with identical 1-16 nucleotides from their 5' ends are combined into one cluster with the most abundant one as the representative. Numbers before the sequences indicate the counts of unique tags and characters in red show mismatches, gaps, and U tails. Red check marks indicate the tags that passed the filter.

Figure S8

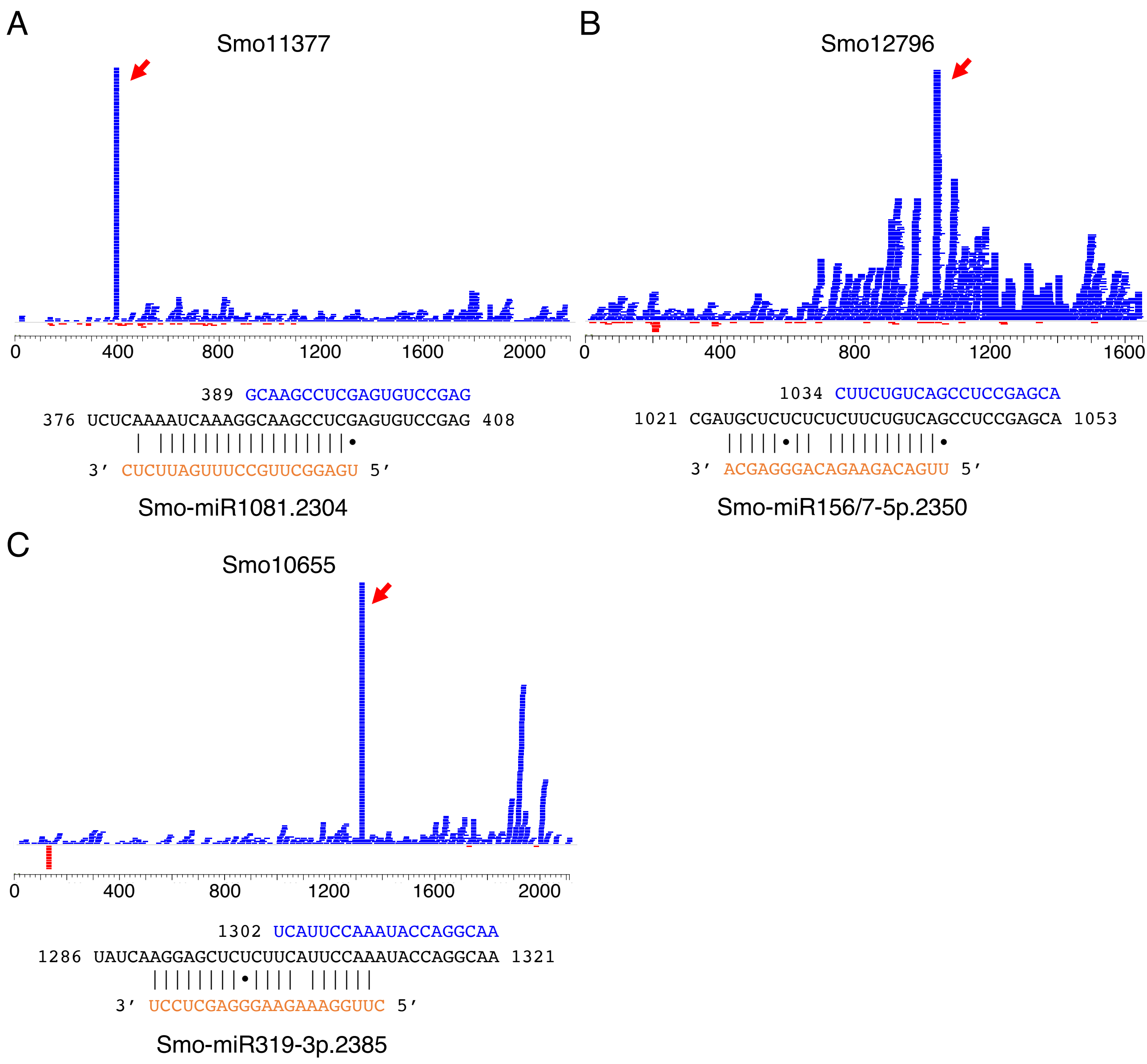
A
Smo11377
0 400 800 1200 1600 2000
389 GCAAGCCUCGAGUGUCCGAG
376 UCUCAAAAUCAAAGGCAAGCCUCGAGUGUCCGAG 408
3′ CUCUUAGUUUCCGUUCGGAGU 5′
Smo-miR1081.2304
B
Smo12796
0 400 800 1200 1600
1034 CUUCUGUCAGCCUCCGAGCA
1021 CGAUGCUCUCUCUCUUCUGUCAGCCUCCGAGCA 1053
3′ ACGAGGGACAGAAGACAGUU 5′
Smo-miR156/7-5p.2350
C
Smo10655
0 400 800 1200 1600 2000
1302 UCAUUCCAAAUACCAGGCAA
1286 UAUCAAGGAGCUCUCUUCAUUCCAAAUACCAGGCAA 1321
3′ UCCUCGAGGGAAGAAAGGUUC 5′
Smo-miR319-3p.2385

Figure S8 Degradome/PARE analyses in *S. moellendorffii*. Three examples of 5' uncapped mRNA fragments and corresponding miRNA candidates that may be responsible for generating these fragments. **(A)** The transcript Smo11377 is likely targeted by Smo-miR1081.2304, which was identified in our small RNA-seq and is identical to annotated smo-miR1081. **(B)** The transcript Smo12796 is likely targeted by Smo-miR156/7-5p.2350. **(C)** The transcript Smo10655 is likely targeted by Smo-miR319-3p. 2385. The horizontal lines with numbers underneath represent the coding regions in the transcripts. The numbers represent the lengths of the coding regions in nucleotides (0 being the “A” of the start codon). Degradome/PARE reads are shown as stacked dashes above the transcript diagrams (blue on the coding strand and red on the complementary strand). The sequences of the miRNA candidates (orange) and the miRNA binding sites (black), as well as those of the 5’ ends of uncapped RNAs called from degradome/PARE sequencing (blue) are shown below the transcript diagrams. Numbers indicate the locations of these sites in the coding region. Perfect pairing between the miRNA and mRNA is indicated by vertical bars, GU pairing is represented by dots, and mismatches are left blank. Red arrows depict the degradome/PARE peaks shown at the nucleotide resolution below.
